# StarNAV: Autonomous Optical Navigation of a Spacecraft by the Relativistic Perturbation of Starlight

**DOI:** 10.3390/s19194064

**Published:** 2019-09-20

**Authors:** John A. Christian

**Affiliations:** Department of Mechanical, Aerospace, and Nuclear Engineering, Rensselaer Polytechnic Institute, Troy, NY 12180, USA; chrisj9@rpi.edu

**Keywords:** StarNAV, autonomous navigation, space exploration, stellar aberration, relativistic Doppler effect, velocity-only orbit determination

## Abstract

Future space exploration missions require increased autonomy. This is especially true for navigation, where continued reliance on Earth-based resources is often a limiting factor in mission design and selection. In response to the need for autonomous navigation, this work introduces the StarNAV framework that may allow a spacecraft to autonomously navigate anywhere in the Solar System (or beyond) using only passive observations of naturally occurring starlight. Relativistic perturbations in the wavelength and direction of observed stars may be used to infer spacecraft velocity which, in turn, may be used for navigation. This work develops the mathematics governing such an approach and explores its efficacy for autonomous navigation. Measurement of stellar spectral shift due to the relativistic Doppler effect is found to be ineffective in practice. Instead, measurement of the change in inter-star angle due to stellar aberration appears to be the most promising technique for navigation by the relativistic perturbation of starlight.

## 1. Introduction

This work presents a method—referred to here as StarNAV—for using perturbations in observed starlight to autonomously navigate a spacecraft in the Solar System. Compared to a reference observer, such as a fictitious stationary observer at the Solar System barycenter (SSB), the starlight measured by a sensor aboard a moving spacecraft will change in both frequency and apparent direction. While there are many phenomena that contribute to these changes, the dominant source for changes in both frequency and apparent direction is due to the Special Theory of Relativity and is explainable within the framework of a Lorentz transformation. The Lorentz transformation, which relates the spacetime coordinates seen by two observers moving relative to one another, depends on the relative velocity between the two observers. Therefore, if relativistic perturbations of starlight in frequency (relativistic Doppler effect) and in apparent direction (stellar aberration) can be measured, these perturbations may be used to infer the spacecraft velocity. In many cases it is possible for a spacecraft to navigate autonomously using only velocity measurements [1,2]. In other cases, such as autonomous navigation with images [3], the addition of direct measurements of velocity may significantly enhance performance.

This work explores two categories of StarNAV measurements: those due to the relativistic Doppler effect (StarNAV-DE measurements) and those due to stellar aberration (StarNAV-SA measurements). These measurement types complement each other, since the Doppler effect (mostly) provides information on the velocity in direction of the observed star while stellar aberration (mostly) provides information on the velocity components in the plane perpendicular to the direction to the observed star. Thus, the velocity-related information content of StarNAV-DE and StarNAV-SA measurements for a single star sighting are (mostly) orthogonal to one another—and together they provide a description of the full three-dimensional (3D) spacecraft velocity. Alternatively, a complete 3D velocity vector may be constructed from just one measurement type (StarNAV-DE or StarNAV-SA) using simultaneous observations of different stars.

Both StarNAV-DE and StarNAV-SA measurement types are now briefly introduced. While the Doppler effect may appear at first to be the most obvious means of obtaining velocity information from starlight, a closer analysis reveals that stellar aberration measurements are almost always preferable.

The spectrum of a star observed by a moving spacecraft is altered by cosmological redshift (due to expansion of the Universe), gravitational redshift/blueshift (due to potential fields near the source and observer), and the relativistic Doppler effect (due to kinematic velocity between the source and observer). Each of these natural phenomena contain numerous contributing effects, with the kinematic relative velocity being the most troublesome due to the active nature of stellar surfaces at short timescales. From a navigation perspective, it is best to separate the effects that may be built into the star’s reference spectrum from the effects that must be considered within the state estimation framework.

Although the concept of using the observed shift of certain spectral lines or of the entire spectrum for navigation is not new—having been considered for navigation within our Solar System [4,5] and beyond [6,7]—many prior studies neglect the essential challenges to such a framework arising from stellar oscillations, granules, and other forms of stellar surface activity (despite many of these challenges being known since nearly the beginning [8]). Indeed, this work demonstrates that autonomous navigation using StarNAV-DE measurements is likely to be ineffective for near-term applications due to instability in the spectral signature of most stars and challenges with instrument calibration. Thus, after a brief development to justify this decision, StarNAV-DE measurements are usually discarded in favor of StarNAV-SA measurements. A few special cases where StarNAV-DE measurements remain competitive (e.g., interstellar flight) are also discussed.

The apparent direction to a star as measured by a moving spacecraft is primarily altered from its nominal star catalog value by (1) the star’s proper motion, (2) parallax, (3) stellar aberration, and (4) the gravitational deflection of light. The first two effects are primarily geometric and are well explained (to the accuracy required here) within a classical Newtonian framework. The latter two effects necessarily require a relativistic framework—with stellar aberration being explainable using only the Special Theory of Relativity and the gravitational deflection of light requiring the General Theory of Relativity. All four of these effects are considered in this work.

As will be shown, the effects of proper motion and parallax can be reduced to below the noise floor required for navigation with information that is readily available in most practical cases. The remaining two effects are both state dependent: stellar aberration is (mostly) a function of the spacecraft velocity and the gravitational deflection of light is (mostly) a function of spacecraft position. The change in apparent star direction from stellar aberration (up to 10 s of arcseconds (arcsec); e.g., ∼26 arcsec in low Earth orbit (LEO)) is generally a few orders of magnitude larger than from the gravitational deflection of light (microarcseonds (μas) to a few milliarcseconds (mas)). Consequently, only the perturbation from stellar aberration is large enough to display usable sensitivity in the vehicle state from the standpoint of autonomous spacecraft navigation. Although the perturbation from the gravitational deflection of light is small and its sensitivity to the spacecraft position is too weak for practical use as a navigation observable (at least with contemporary sensors), the effect is still large enough that it must be explicitly accounted for in any reasonable navigation system. The gravitational deflection of light manifests itself as a small bias, which can usually be estimated by a single scalar parameter.

When collected with suitable accuracy, stellar aberration measurements (i.e., StarNAV-SA measurements) permit the direct estimation of the vehicle’s inertial velocity which, in turn, may be used for navigation. The fundamental concept of using stellar aberration for autonomous navigation was also suggested in [9]. However, this earlier work presented only a cursory analysis, considered only first-order stellar aberration effects (despite requiring measurement accuracy corresponding to third-order relativistic effects), and neglected entirely the gravitational deflection of starlight (which is also orders of magnitude larger than the required measurement accuracy). The idea appears to have been largely lost with the passing of time, but is given new life by recent advancements in velocity-only orbit determination [2], an elegant mathematical framework for microarcsecond astrometry [10], and improved astrometric data in modern star catalogs [11]. This work provides evidence that the StarNAV-SA approach is a feasible means of autonomous navigation that may offer an alternative (or a complement to) other autonomous navigation systems under development today (e.g., X-ray Pulsar Navigation (XNAV)).

This work shows that navigation performance suitable for many common mission types may be achieved when star directions are measured with errors on the order of 0.1–1 mas. Particular mission scenarios—in Earth orbit or elsewhere—may require better or worse sensor performance. Hence, undue importance should not be ascribed to the choice of 0.1–1 mas of sensor error used throughout this work; it is a reasonable number for the preliminary analysis of the StarNAV framework and nothing more.

The remainder of this work is organized as follows: Section 2 provides background and historical context. Section 3 develops mathematical models for the underlying natural phenomena. Section 4 considers StarNAV feasibility by considering how well the natural phenomena outlined in Section 3 may be measured in practice. The remaining sections consider the efficacy of StarNAV for navigation: Section 5 considers an instantaneous velocity fix, Section 6 considers initial orbit determination (IOD), and Section 7 considers real-time navigation with an extended Kalman filter (EKF).

## 2. Background

### 2.1. Need for Autonomous Spacecraft Navigation

Autonomous spacecraft navigation is an enabling technology for a wide variety of future spaceflight missions, ranging from LEO constellations to interplanetary science missions. Low-cost autonomous navigation in LEO is made possible by the prevalence of global navigation satellite systems (GNSS), such as the United States’ Global Positioning System (GPS) or the European Union’s Galileo. However, there is sometimes a need for autonomous navigation capability in LEO when GNSS is unavailable. There is also a need for autonomous navigation when traveling to destinations beyond LEO (e.g., Earth’s moon, other planets, asteroids, comets, Earth-Sun libration points) where no GNSS-type observables exist.

These demands for autonomous navigation have led to substantial investment in autonomous navigation technologies in recent decades. Three such technologies are highlighted here as illustrative examples. First is XNAV, which uses the time-of-arrival of pulses from stable millisecond pulsars to estimate the spacecraft’s position [12,13,14]. Real-time, on-board XNAV was recently demonstrated with the SEXTANT experiment [15,16] on the International Space Station (ISS). Second is classical spacecraft optical navigation (OPNAV), which uses images of nearby celestial bodies against a star field background. Autonomous OPNAV with horizon-based methods [3] works well with resolved imagery of ellipsoidal bodies and will be demonstrated on NASA’s Artemis 1 mission [17]. Autonomous OPNAV with unresolved imagery (e.g., of asteroids [18,19] or moons [20]) may be used for kinematic positioning (essentially triangulation), a procedure that was demonstrated using JPL’s AutoNav system on the Deep Space 1 mission [21]. Third is enabling one-way ranging using radio frequency (RF) signals from the Deep Space Network (DSN) through the Deep Space Atomic Clock (DSAC) [22,23,24], which was launched during the writing of this manuscript (June 2019).

### 2.2. Remarks on the History of Star-Based Navigation

Celestial navigation and the practice of using star sightings for navigation has existed since antiquity [25,26]. The utility of stars for navigation must have been plainly obvious, as similar techniques were independently developed by European [27], Arabian [28], Chinese [29], and Polynesian [30] explorers. The earliest explorers—on both land and sea—are known to have navigated by observing the elevation of the Pole Star and by memorizing the location of guide stars as they rose or set. With time and the advent of written language, memorization gave way to carefully curated star charts and tables [31]. Meanwhile, star sightings were collected with ever improving instrumentation [27] (e.g., astrolabe [32,33], sextant [34]). Expert navigators trained in the arts of cartography, astrometry, and chronometry became essential members of all types of expeditions (e.g., exploration, scientific, mercantile), especially on the open sea where there were no roads or geographic features to follow. The practice of manual celestial navigation remained a critical skill for the professional mariner until it was largely replaced with global navigation satellite systems (GNSS) in the late twentieth century. While the proliferation of GNSS-based navigation systems has led to the complete abandonment of celestial navigation in many sectors, celestial navigation still enjoys support in niche applications and has seen notable mathematical and technological advances in the past 25 years [35,36,37,38,39].

Given humanity’s long history of navigation by stars, it comes as no surprise that some of the first methods for autonomous spacecraft navigation relied on astronauts manually taking star sightings with a space sextant [40,41,42,43,44]. While this practice continues to modern day (e.g., a new space sextant is currently under development for future human space exploration [45]), the prevalence of robotic (uncrewed) spacecraft provided ample motivation to automatically collect celestial navigation measurements with a camera. The use of images of celestial bodies against a starfield background—generally referred to as optical navigation (OPNAV)—has been studied extensively [3,46] and has been a critical component of most outer planet exploration missions [47,48,49,50,51,52].

Conventional star sightings—either manually with a sextant or automatically with a camera—fundamentally provide information about an inertially fixed reference direction. Positional information (e.g., estimation of a ship’s latitude or a spacecraft’s orbit) comes not from the star direction, but the observed direction of a feature belonging to a foreground celestial object (e.g., the Earth’s horizon) relative to the inertially fixed star field. These relative directions (which form an angle), together with time and a model of the celestial body’s motion, permit estimation of the observer’s location. Therefore, images of star fields with no foreground body, such as those acquired by a star tracker [53,54], are generally used for attitude estimation only [55].

Modern (circa 2019) star trackers and OPNAV cameras are capable of determining attitude with errors on the order of 1–10 arcsec [56,57,58]. Estimation of attitude at this level requires the sensor system be supplied with the vehicle’s inertial velocity (relative to the star catalog’s reference frame) to remove the attitude bias introduced by stellar aberration [59], which can amount to about 26 arcsec for a spacecraft in LEO. The stellar aberration generally manifests itself as an attitude bias because most star trackers have a relatively narrow field-of-view (FOV) and all the observed stars experience a similar perturbation. Consequently, the vehicle’s inertial velocity and the resulting attitude bias from stellar aberration are a nuisance parameter that must be compensated for to achieve state-of-the-art pointing knowledge. In contrast to the conventional use of stars for navigation (or attitude determination), this work follows the suggestion of [9] and takes the nuisance parameter of stellar aberration and turns it into the navigation observable.

While spacecraft navigation using star bearing measurements has been practiced since the very first days of spaceflight, navigation by the shift in stellar (or solar) spectra due to the Doppler effect has only been suggested and (to the author’s knowledge) never implemented in practice. While the possibility of spacecraft navigation by the Doppler shift of spectra was recognized by at least the early 1960s [4], the practical difficulties in such an approach were quickly realized [8]. Despite these challenges, numerous studies were subsequently performed on the efficacy of navigation using Doppler shift from the Sun [60,61,62] and from stars [5,63]—with some authors (e.g, [62]) ultimately abandoning the approach for reasons similar to [8]. Of note is that many of these complications may be avoided within the context of relative navigation by comparing the spectra observed at more than one location, thus, allowing one to estimate the relative velocity between the two observers. Applications of this approach include formation flying [64] or orbit determination by reflected sunlight [65,66,67]. However, the comparison of simultaneously recorded spectra at two different locations is not compatible with the philosophy of autonomous navigation and is not explored further in this work.

Finally, discussion of both stellar aberration and the relativistic Doppler effect as navigation observables also appears in the few serious papers that exist on the topic interstellar navigation (e.g., [6,68]), although detailed analysis appears to be lacking in the published record of these works. As with conventional celestial navigation, many interstellar navigation studies still consider stellar aberration as a nuisance parameter to be corrected instead of a navigation observable in its own right [69,70,71]. Autonomous interstellar navigation concepts generally estimate velocity exclusively by the relativistic Doppler effect [6,7] and rarely address the practical challenges associated with the stability stellar spectra. There are examples of concept studies that do use stellar aberration to estimate velocity (e.g., [72]), but these appear to be few in number.

## 3. Mathematical Models for the Observation of Starlight by a Moving Spacecraft

### 3.1. Reference Star Models

If one is to use the relativistic perturbation of starlight to determine the observer (spacecraft) velocity, it is first necessary to have an accurate model of star spectra and directions in the absence of such effects. These models are generally referenced to a fictitious stationary observer at the SSB in the absence of general relativistic perturbations from Solar System’s potential field (i.e., at zero potential).

Models for star direction make use of data tabulated in star catalogs that are painstakingly constructed from astrometric observations. Likewise, models of star spectra may be cataloged. Further, in addition to directions and spectra, models for stellar photon flux are essential for understanding sensor performance. The following subsections address key aspects of these three reference models (directions, spectra, photon flux).

#### 3.1.1. Star Catalogs and Astrometric Models

Astrometry—the branch of astronomy concerned with measuring the position and motion of celestial objects—is one of the oldest known branches of the physical sciences [73]. Indeed, astrometric data has long been recorded in star catalogs to capture our best knowledge of star locations [31,74]. The present work assumes the use of modern catalogs as exemplified by the Hipparcos Catalog [75,76] and Gaia Catalog [11,77], which were produced by European Space Agency (ESA) space astrometry missions of the same name [78,79]. At the time this paper was written, the state-of-the-art is best represented by the Gaia Data Release 2 [77], whose astrometric data can produce star line-of-sight (LOS) unit vectors with bearing errors below 0.1 mas [11].

Modern catalogs generally store astrometric data with respect to a particular realization of the International Celestial Reference Frame (ICRF) [80,81,82,83] with the origin shifted to the SSB—defined as the Barycentric Celestial Reference Frame (BCRF). This is the case for the Gaia Data Release 2 used in this study [11], as well as for many other contemporary catalogs.

For consistency with the available catalog data, this work adopts the same “standard model” for star directions as employed for the reduction of both Hipparcos and Gaia observations [84]. This is a six-parameter model considering the following parameters for each (*i*-th) star at the time of the catalog epoch ($t_{ep}$): BCRF right ascension ($\alpha_{i}$), BCRF declination ($\delta_{i}$), proper motion in right ascension direction ($\mu_{{\alpha *}_{i}}$), proper motion in declination direction ($\mu_{\delta_{i}}$), radial proper motion ($\mu_{r_{i}}$), and annual parallax ($\varpi_{i}$). While other models and parameterizations may be chosen, the present work is bound to this standard model since the use of either the Hipparcos or Gaia catalogs is presumed. Reformulation of the following with other reasonable parameterizations is straightforward and (if necessary) left as an exercise to the reader.

The six-parameter model is defined as follows. Let the right ascension and declination be used to define the star LOS unit vector to the *i*-th star as seen by an observer at the BCRF origin (solar system barycenter) at the catalog epoch ($t_{ep}$), denoted as $\ell_{i}$, such that

(1) $$\ell_{i}=\left[\begin{array}{c} \cos\left(\delta_{i}\right)\cos\left(\alpha_{i}\right)\\ \cos\left(\delta_{i}\right)\sin\left(\alpha_{i}\right)\\ \sin(\delta_{i}) \end{array}\right]$$

Further define the orthogonal unit vectors (where $z^{T}=\left[0\;0\;1\right]$)

(2) $$p_{i}=\langle z\times \ell_{i}\rangle =\left[\begin{array}{c} -\sin(\alpha_{i})\\ \cos(\alpha_{i})\\ 0 \end{array}\right]\;\;\;\;\;q_{i}=\ell_{i}\times p_{i}=\left[\begin{array}{c} -\sin\left(\delta_{i}\right)\cos\left(\alpha_{i}\right)\\ -\sin\left(\delta_{i}\right)\sin\left(\alpha_{i}\right)\\ \cos(\delta_{i}) \end{array}\right]$$

such that the ordered triad $\left\{p_{i},q_{i},\ell_{i}\right\}$ forms a right-handed orthonormal basis for $R^{3}$. The triangular brackets $\langle \cdot \rangle$ denote vector length normalization (i.e., $\langle x\rangle =x/∥x∥$ ). From here, the six-parameter model for $u_{i}$ (where $u_{i}$ is the LOS unit vector to the *i*-th star as seen by an observer at BCRF position r(t) at observation time *t*) may be written directly in terms of $\ell_{i}$ and the *i*-th star’s remaining four astrometric parameters as [84]

(3) $$u_{i}\left(t\right)=\langle \ell_{i}+\left[t+\ell_{i}^{T}r\left(t\right)/c-t_{ep}\right]\left(\mu_{{\alpha *}_{i}}p_{i}+\mu_{\delta_{i}}q_{i}+\mu_{r_{i}}\ell_{i}\right)-\varpi_{i}r\left(t\right)/A_{u}\rangle$$

where c=299,792,458 m/s is the speed of light and $A_{u}=149,597,870,700$ m is the astronomical unit (AU).

The contribution from the radial velocity is negligible for all but a small number of stars, leading to the assumption of $\mu_{r_{i}}=0$ for the reduction of most Gaia data in practice [11]. Thus, the full six-parameter model from Equation (3) becomes the usual five-parameter model,

(4) $$u_{i}\left(t\right)=\langle \ell_{i}+\left[t+\ell_{i}^{T}r\left(t\right)/c-t_{ep}\right]\left(\mu_{{\alpha *}_{i}}p_{i}+\mu_{\delta_{i}}q_{i}\right)-\varpi_{i}r\left(t\right)/A_{u}\rangle$$

**Figure 1:**
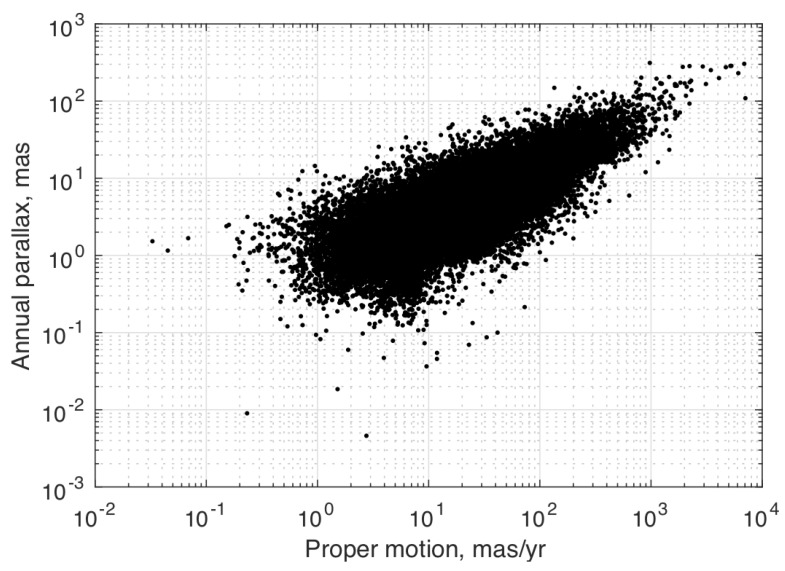
Distribution of proper motion magnitude and annual parallax for stars with magnitude G≤8 from Gaia Data Release 2. For raw data see [77].

Furthermore, observe that the term $\ell_{i}^{T}r\left(t\right)/c$ (the so-called Roemer delay) has a worst-case value on the order of 500 s at 1 AU. Assuming the median proper motion magnitude of 21.3 mas/year from the Gaia catalog (for G≤8, see Figure 1), this amounts to only a 0.34 μas modification of the LOS direction. Consequently, this effect can be safely neglected in most cases, leading to

(5) $$u_{i}\left(t\right)=\langle \ell_{i}+\left(t-t_{ep}\right)\left(\mu_{{\alpha *}_{i}}p_{i}+\mu_{\delta_{i}}q_{i}\right)-\varpi_{i}r\left(t\right)/A_{u}\rangle$$

The only remaining state-dependent term in Equation (5) is the one describing parallax. The median annual parallax from the Gaia catalog for a spacecraft in Earth orbit is 3.9 mas (for G≤8, see Figure 1), which is clearly of a magnitude that cannot be neglected. Fortunately, however, this effect is driven by the BCRF position. Consequently, for a vehicle in orbit about a planet, the planet’s ephemeris (which is known) will remove the vast majority of this effect. For example, the median residual parallax for a spacecraft in geostationary orbit (GEO) is about 1 μas. Thus, for a spacecraft orbiting a known celestial body, the star LOS direction may often be approximated to a suitable accuracy by

(6) $$u_{i}\left(t\right)=\langle \ell_{i}+\left(t-t_{ep}\right)\left(\mu_{{\alpha *}_{i}}p_{i}+\mu_{\delta_{i}}q_{i}\right)-\varpi_{i}r_{B}\left(t\right)/A_{u}\rangle$$

where $r_{B}\left(t\right)$ is the BCRF position of the celestial body the spacecraft is orbiting.

For a vehicle in heliocentric orbit far away from a planet, the BCRF position may be autonomously determined to sufficient accuracy to remove parallax (∼$10^{5}$ km) through triangulation using optical LOS observations of known planets [85], asteroids [19], or both.

#### 3.1.2. Models for Reference Stellar Spectra

Stellar spectra are one of the principal observational data types used in modern astronomy and astrophysics. As with star directions, stellar spectra have long been cataloged [86]. Of particular relevance here, the shifts in stellar spectra are often used to estimate radial velocity—a critical ingredient in the fields of astrometry, astroseismology, and the search for exoplanets.

The naive application of the classical Doppler equation may lead the well-meaning navigator to erroneously suggest that the inertial velocity can be related to the observed frequency shift in stellar spectra according to

(7) $$\frac{u^{T}v}{c}=\frac{\Delta f}{f_{0}}$$

where u is the unit vector from the observer to the source, $f_{0}$ is the frequency seen by an observer at rest, and Δf is the change in frequency seen by an observer moving at velocity v relative to the rest frame. Such an approach, however, introduces a few m/s of error at best and up to a few hundred m/s of error at worst. A more careful analysis is clearly required.

To begin, a more precise definition of the frequency shift is required, as multiple conventions exist in the literature and their difference is important at the level of precision required here. This work adopts the following definition for a spectral shift, *z*,

(8) $$z=\frac{\lambda -\lambda_{0}}{\lambda_{0}}=\frac{f_{0}-f}{f}$$

where $\lambda_{0}$ (or $f_{0}$) is the reference wavelength (or frequency) and λ (or *f*) is the measured wavelength (or frequency) seen by an observer moving relative to the reference. Therefore, defining $\Delta \lambda =\lambda -\lambda_{0}$ and $\Delta f=f-f_{0}$, one obtains

(9) $$z=\frac{\Delta \lambda }{\lambda_{0}}=-\frac{\Delta f}{f}$$

Here, an important distinction is made between Δf/f and $\Delta f/f_{0}$,

(10) $$\frac{\Delta f}{f}=-z\;\mathrm{vs}.\;\frac{\Delta f}{f_{0}}=\frac{-z}{1+z}$$

such that it matters if the ratio of Δf is taken with respect to *f* or $f_{0}$, with this choice leading to a difference of $O(z^{2})$ between the two.

The frequency of starlight is altered by numerous sources between its origin within a star and when it is received by an instrument (e.g., spectrograph) on a spacecraft within our Solar System. These frequency shifts are due to three principal phenomena: cosmological redshift, gravitational redshift/blueshift, and the relativistic Doppler effect. Cosmological redshift is due to the expansion of the Universe, causing apparent stretching of the wavelength when the source and observer are far apart. Gravitational redshift occurs as the light escapes the potential field of the source star, while gravitational blueshift occurs as the light falls into the potential field of our Solar System where the spacecraft resides. Finally, change in frequency occurs from the relativistic Doppler effect due to the kinematic velocity between the source star and the spacecraft. This relative velocity is due to a number of sources, including: (1) the velocity of the spacecraft relative to the SSB, (2) the velocity of the SSB relative to the observed star’s barycenter, (3) velocity of the star center-of-mass relative to its barycenter induced by the gravitational attraction of its planets, (4) activity on the surface of the star, and (5) rotational motion of the asymmetric star. To make a usable navigation system, one must ultimately isolate Doppler effect due to source #1 (the velocity of the spacecraft relative to the SSB) from all the other contributing phenomena and Doppler effect sources. This is critical, since nearly every item listed here can contribute errors at the m/s level (or higher), depending on the particular star.

The chain of frequency shifts described above make it impossible to determine the true kinematic radial velocity (at the m/s level or better) between a source star and an observer in our Solar System purely from measurements of the star’s spectrum [87]. Fortunately, the navigation problem only requires one to know the relative velocity between the observer (spacecraft) and the SSB. In practice, this would require a reference spectrum at the SSB to be created for each StarNAV-DE guide star.

Expressing stellar spectra at the SSB is common practice within the scientific community, where an observed spectrum may be transformed to the SSB with zero potential by (1) removing the gravitational blueshift from the observer’s location within the Solar System’s potential field and (2) removing the Doppler effect from the observer’s velocity relative to the SSB. Therefore, define the frequency at the source star as $f_{*}$, the frequency at the SSB with zero potential as *f*, the frequency of a fictitious stationary observer at the spacecraft’s instantaneous position as $f^{\prime }$, and the frequency seen by an observer onboard the moving spacecraft as $f^{\prime \prime }$. The chain of frequency shifts is then

(11) $$f_{*}=\left(\frac{f_{*}}{f}\right)\left(\frac{f}{f^{\prime }}\right)\left(\frac{f^{\prime }}{f^{\prime \prime }}\right)f^{\prime \prime }$$

where the individual contributing factors can be written in terms of frequency shifts as

(12) $$z_{B}=\frac{f_{*}-f}{f}\;\to \;\frac{f_{*}}{f}=z_{B}+1$$

(13) $$z^{\prime }=\frac{f-f^{\prime }}{f^{\prime }}\;\to \;\frac{f}{f^{\prime }}=z^{\prime }+1$$

(14) $$z^{\prime \prime }=\frac{f^{\prime }-f^{\prime \prime }}{f^{\prime \prime }}\;\to \;\frac{f^{\prime }}{f^{\prime \prime }}=z^{\prime \prime }+1$$

such that

(15) $$f_{*}=\left(z_{B}+1\right)\left(z^{\prime }+1\right)\left(z^{\prime \prime }+1\right)f^{\prime \prime }$$

Therefore, when building a reference spectrum for a star, one may collect observations of $f^{\prime \prime }$ over a long period of time to compute a time-history of *f* according to

(16) $$f=\left(z^{\prime }+1\right)\left(z^{\prime \prime }+1\right)f^{\prime \prime }$$

where $\left(z^{\prime }+1\right)$ removes the gravitational blueshift and $\left(z^{\prime \prime }+1\right)$ removes the Doppler shift of the observer’s motion relative to the SSB. The shifts $z^{\prime }$ and $z^{\prime \prime }$ are generally well-known when building a reference spectrum from scientific observations. At a high-level, this process is reversed for navigation with StarNAV-DE measurements, when *f* and $f^{\prime \prime }$ are known and one solves for the spacecraft state information embedded in the shifts $z^{\prime }$ and $z^{\prime \prime }$. Mathematical development of $z^{\prime }$ and $z^{\prime \prime }$ may be found in Section 3.3.

This time-history of *f* generated from Equation (15) will have oscillations at many different timescales that are often the focus of scientific study. From the standpoint of navigation, however, these oscillations are a nuisance (see Section 4.2) and the present work assumes a long-term average for *f* is generated.

Note, of course, that the shifted frequency *f* is related to what one might otherwise expect (e.g., from the location of absorption lines for specific elements) according to

(17) $$f_{*}=\left(z_{B}+1\right)f$$

This frequency shift may be written as a speed (instead of as a dimensionless frequency ratio) by multiplying the SSB shift $z_{B}$ by the speed of light. Doing so results in the so-called “barycentric radial-velocity measure,” $cz_{B}$, which is a standardized astrometric parameter often used to describe the shift of the underlying spectral model to the SSB. A detailed discussion about computing $cz_{B}$ is provided in [87].

#### 3.1.3. Models for Stellar Photon Flux

Models of stellar photon flux are essential in evaluating the performance of any star-observing system. In the case of StarNAV, measuring either the direction to or spectra of a star critically relies on having a sufficient number of photons available for the sensor to detect. From a design standpoint, it is useful to express the photon flux as a function of star magnitude (and, perhaps, star type), as this information is readily available in most star catalogs.

This task is somewhat complicated by the different systems of star magnitude in use today [88]. Unless otherwise specified, this work assumes magnitudes are defined using the Johnson-Cousins system [89,90]. Transformation to other photometric systems—such as the Sloan Digital Sky Survey system [91] or the Gaia system [92]—is straightforward and left to the reader. This work exclusively uses apparent magnitudes and all references to *magnitude* are assumed to be *apparent magnitude*. Regardless of the specific convention, most photometric magnitude systems describe the apparent irradiance (*E*) relative to a reference irradiance ($E_{0}$) on a logarithmic scale,

(18) $$m=-2.5\log_{10}\frac{E}{E_{0}}$$

where the zero point is arbitrary and chosen by convention, often with zero magnitude being defined as approximately that of Vega (α Lyr). Vega has a visual magnitude of 0.03 in the Johnson-Cousins system [88].

The objective now is to relate photon flux to star magnitude. In practice, the usefulness of a photon flux model is fundamentally related to the sensor’s spectral sensitivity. Given a spectral irradiance of E(λ), the irradiance measured by a sensor in space with spectral sensitivity S(λ) is [88,93]

(19) $$E_{S}=\int E\left(\lambda \right)S\left(\lambda \right)d\lambda$$

and the corresponding photon flux is

(20) $$n_{S}=\frac{1}{hc}\int \lambda E\left(\lambda \right)S\left(\lambda \right)d\lambda$$

where $h=6.62607015\times 10^{-34}$ J·s is the Planck constant and *c* is the speed of light. When S(λ) is the passband for the Johnson-Cousins V-band filter, one obtains the measured irradiance ($E_{V}$) and photon flux ($n_{V}$) corresponding to the visual magnitude $m_{V}$. Given the conventions of the Johnson-Cousins system, a star of visual magnitude $m_{V}=0$ has a spectral irradiance at the V-band effective wavelength of $E\left(\lambda_{V}\right)\approx 3.64\times 10^{-23}$ W/m${}^{2}$/Hz [94].

As a useful reference, temporarily assume that all photons are received at the V-band’s effective wavelength, $\lambda_{V}=544.8$ nm [88]. Recognizing that the V-band filter has a passband (full width half maximum) of around Δλ≈84 nm [88] and that $∥\Delta f∥\approx c∥\Delta \lambda ∥/\lambda^{2}$, the idealized photon flux for a $m_{V}=0$ star may be found by approximating the integral from Equation (20) as

(21) $$n_{V_{0}}\approx \frac{\lambda_{V}}{hc}\frac{c\Delta \lambda }{\lambda_{V}^{2}}E\left(\lambda_{V}\right)=\frac{1}{h}\frac{\Delta \lambda }{\lambda_{V}}E\left(\lambda_{V}\right)=8.47\times 10^{9}\;\mathrm{photons}/m^{2}/\mathrm{second}$$

which may be written in exponential form as $n_{V_{0}}\approx 10^{9.93}$ photons/m${}^{2}$/second. This is generally consistent with the value of $n_{V_{0}}\approx 10^{9.94}$ photons/m${}^{2}$/second presented in [93]. Consequently, given the definition of star magnitude from Equation (18), the V-band photon flux for a star of apparent visual magnitude $m_{V}$ is given by

(22) $$n_{V}=\left(8.47\times 10^{9}\right)\left(10^{-0.4m_{V}}\right)\;\mathrm{photons}/m^{2}/\mathrm{second}$$

As noted above, stars do not emit all their photons at one wavelength. Furthermore, many optical sensors have a spectral sensitivity that captures more photons than what is passed by the Johnson-Cousins V-band filter, such that the photon flux seen for a $m_{V}$ star often exceeds that of Equation (22) in practice. These issues are now addressed using the above results as a reference.

Stars are generally well-modeled as blackbody radiators, such that the spectral irradiance of a star with temperature *T* is proportional to the distribution described by Planck’s law [95],

(23) $$E\left(\lambda \right)\propto W\left(\lambda ,T\right)=\frac{2hc^{2}}{\lambda^{5}}{\left[\exp\left(\frac{hc}{\lambda kT}\right)-1\right]}^{-1}$$

where $k=1.380649\times 10^{-23}$ J/K is the Boltzmann constant. Consequently,

(24) $$\frac{E(\lambda_{1})}{E(\lambda_{2})}=\frac{W(\lambda_{1},T)}{W(\lambda_{2},T)}$$

Thus, if one defines $\hat{W}\left(\lambda ,T\right)$ to be the ratio

(25) $$\hat{W}\left(\lambda ,T\right)=\frac{W(\lambda ,T)}{W(\lambda_{V},T)}$$

then, by rearrangement of Equation (20), it is relatively straightforward to show that [93]

(26) $$n=n_{V}\left[10^{1.05}\int D\left(\lambda \right)\left(\frac{\lambda }{\lambda_{V}}\right)\hat{W}\left(\lambda ,T\right)d\lambda \right]$$

where D(λ) is the spectral sensitivity of the detector. The value for the integral depends on the detector of choice and temperature of the observed star. Values of this integral are tabulated in [93] for many different combinations of detector type and star type. Assuming the detector is a charged coupled device (CCD) [96] and the observed star is of type F or G, the bracketed term in Equation (26) evaluates to [93]

(27) $$10^{1.05}\int D\left(\lambda \right)\left(\frac{\lambda }{\lambda_{V}}\right)\hat{W}\left(\lambda ,T\right)d\lambda \approx 10^{0.51}=3.24$$

Thus, substitution of Equations (22) and (27) into Equation (26) leads to the expression for the photon flux from a F or G type star of magnitude $m_{V}$ that would be seen with a typical CCD detector

(28) $$n\approx \left(2.741\times 10^{10}\right)\left(10^{-0.4m_{V}}\right)\;\mathrm{photons}/m^{2}/\mathrm{second}$$

The resulting photon flux from Equation (28) is tabulated in Table 1 for stars of magnitude 0 to 6. Compared with a F or G star of the same magnitude, stars of most other types (e.g., A, B, K) will generally appear to have a slightly higher photon flux due to the spectral sensitivity of most CCDs—thus, the result of Equation (28) is a conservative estimate.

### 3.2. Perturbations in Apparent Direction of Starlight

#### 3.2.1. Gravitational Deflection of Starlight in the Solar System

The gravitational deflection of light is one of the many consequences of general relativity. Such deflection comes from a number of different sources (as discussed at length in [10]), each of which may be considered separately. The main sources include starlight deflection from: (1) spherically symmetric part of gravity field of large bodies in the Solar System, (2) non-symmetric part of bodies’ gravity field, (3) gravitomagnetic field induced by bodies’ translational motion, and (4) gravitomagnetic field induced by bodies’ rotational motion. Assuming that star observations are not taken at grazing angles to the Sun or to any of planets (especially Jupiter), only the spherically symmetric gravity field effects are important at the levels of measurement accuracy considered here [10,98].

Consider a model for the gravitational deflection of starlight of the form

(29) $$u_{i}^{\prime }=u_{i}+\delta u_{i}$$

where $u_{i}$ is the unit vector describing the direction to the *i*-th star in the absence of (or infinitely far from) the gravitating bodies as produced by the model of appropriate accuracy from Section 3.1.1. Furthermore, let $u_{i}^{\prime }$ be the unit vector describing the direction to the *i*-th star as seen by a fictitious observer at BCRF position r with zero velocity (the velocity effects are considered later as stellar aberration).

Define the distance from the spacecraft to any one of the gravitating bodies as

(30) $$\rho_{B}=∥r_{B}-r∥$$

such that the unit vector describing the direction from the spacecraft to the body is

(31) $$u_{B}=\langle r_{B}-r\rangle =\frac{r_{B}-r}{\rho_{B}}$$

**Figure 2:**
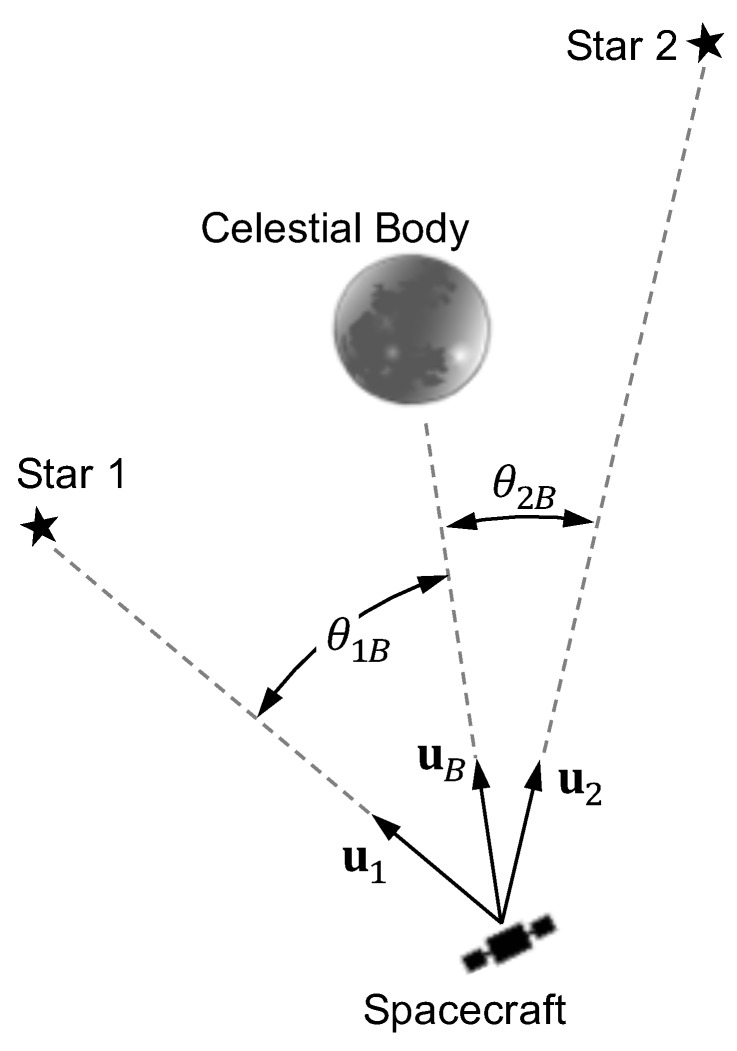
Geometry of stars and celestial bodies for computation of the gravitational deflection of light.

Further define the angle between the *i*-th star and one of the gravitating bodies as seen by the spacecraft (Figure 2) as

(32) $$\cos\left(\theta_{iB}\right)=u_{i}^{T}u_{B}$$

Under these conditions, the deflection of starlight due to the spherically symmetric gravity fields of the Solar System bodies is given by [10]

(33) $$\delta u_{i}=-\sum_{B}\frac{\left(1+\gamma_{PPN}\right)GM_{B}}{c^{2}{∥d_{iB}∥}^{2}}\left(1+u_{i}^{T}u_{B}\right)d_{iB}$$

where $G=6.674\times 10^{-11}\;m^{3}{\mathrm{kg}}^{-1}s^{-2}$ is the universal gravitational constant and $M_{B}$ is the mass of celestial body *B*. The scalar $\gamma_{PPN}$ is one of the principal parameters within the parameterized post-Newtonian (PPN) formalism [99,100,101]. Under general relativity $\gamma_{PPN}$ is equal to unity. One of the best estimates of $\gamma_{PPN}$ to date is due to radiometric tracking of the Cassini spacecraft with DSN [102], which found that $\gamma_{PPN}-1=\left(2.1\pm 2.3\right)\times 10^{-5}$. Hence, one expects $\left(1+\gamma_{PPN}\right)\approx 2$.

The 3×1 vector $d_{iB}$ is defined as

(34) $$d_{iB}=u_{i}\times \left[\left(r_{B}-r\right)\times u_{i}\right]=-{\left[u_{i}\times \right]}^{2}\left(r_{B}-r\right)=\left(I_{3\times 3}-u_{i}u_{i}^{T}\right)\left(r_{B}-r\right)$$

where $\left[\cdot \times \right]$ is the skew-symmetric cross product matrix, such that $a\times b=\left[a\times \right]b$. Observe that $d_{iB}$ (for each celestial body) and $\delta u_{i}$ lie in the plane perpendicular to $u_{i}$ by construction.

Therefore, assuming $(1+\gamma_{PPN})=2$, the magnitude of the gravitational deflection described in Equation (33) for any given body may be computed as [10,100]

(35) $$\delta u_{iB}=\frac{2GM_{B}}{c^{2}\rho_{B}}\frac{1+\cos\left(\theta_{iB}\right)}{\sin\left(\theta_{iB}\right)}=\frac{2GM_{B}}{c^{2}\rho_{B}}\cot\left(\theta_{iB}/2\right)$$

which is applied in the direction $w_{iB}$

(36) $$w_{iB}=\langle d_{iB}\rangle =\langle \left(I_{3\times 3}-u_{i}u_{i}^{T}\right)u_{B}\rangle$$

such that Equation (33) may also be written as

(37) $$\delta u_{i}=-\sum_{B}\delta u_{iB}\;w_{iB}$$

**Figure 3:**
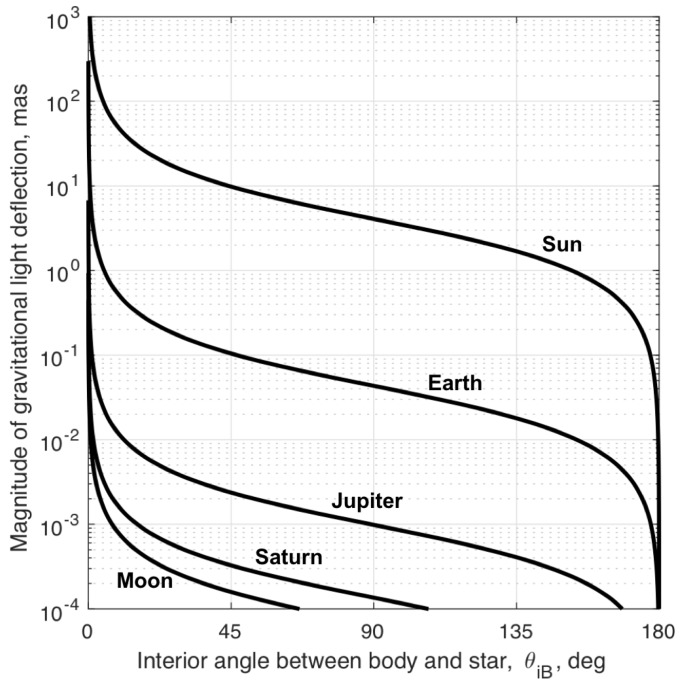
Magnitude of gravitational deflection of starlight from the spherical gravity field of the five most significant celestial bodies for a spacecraft in geostationary orbit.

**Figure 4:**
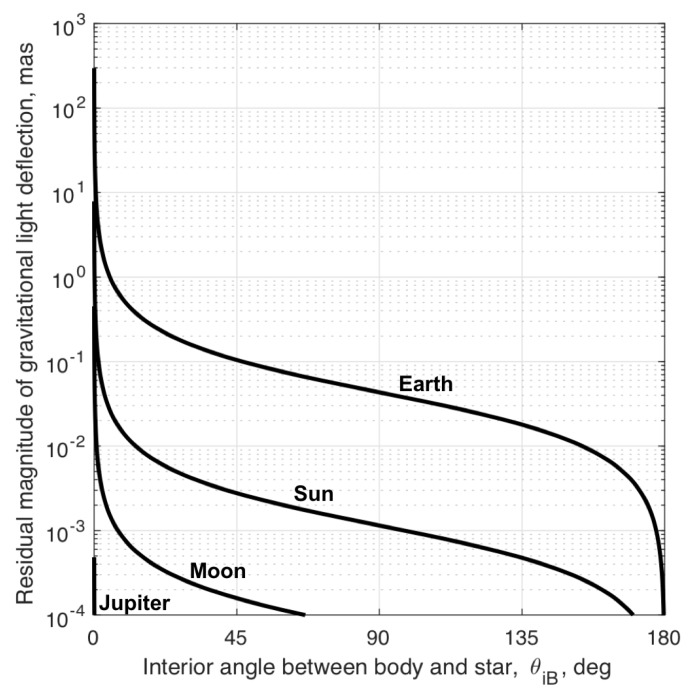
Residual magnitude of gravitational deflection of starlight from the spherical gravity field of the five most significant celestial bodies for a spacecraft in geostationary orbit. Entirety of line for Saturn is below $10^{-4}$ mas.

In general, the magnitude of the gravitational deflection of starlight is large enough that it must be explicitly accounted for within the StarNAV framework. While it is always necessary to consider deflection from the Sun for missions within the Solar System, which additional terms are important depends greatly on the specific mission scenario. As an illustrative example, consider a spacecraft in geostationary orbit. The magnitude of the gravitational deflection of starlight as described by Equation (35) for such an example is shown in Figure 3. These curves reflect the worst-case deflection induced by the five most significant bodies in the Solar System. The results show that the gravitational deflection of light must be considered for the Earth and Sun in almost all cases, while Jupiter and Saturn need only be considered for star sightings very near those planets (small $\theta_{iB}$). Fortunately, with the exception of the Earth and Moon, the majority of the light deflection comes from the relative position between Earth and the other gravitating body (i.e., since the spacecraft is in Earth orbit $∥r-r_{B}∥\approx ∥r_{E}-r_{B}∥$). Therefore, presuming the spacecraft is known to be in Earth orbit, one may assume the BCRF position of the spacecraft is approximately the BCRF position of Earth ($r\approx r_{E}$) for the gravitational deflection induced from all bodies other than the Earth and Moon. In this case, the residual deflection of starlight is as shown in Figure 4.

These results may be used to develop exclusion angles that guarantee the deflection of light by a particular body remains below a specified threshold. Body exclusion angles for the geostationary example discussed here are shown in Table 2. As with Figure 3 and Figure 4, the exclusion angles are produced by the evaluation of Equation (35). Assuming a sensor capable of measuring an inter-star angle to within 0.1 mas, the threshold for exclusion is set to a starlight deflection of 0.01 mas (one order of magnitude smaller than the measurement noise). Two exclusion angles are shown in Table 2. The first exclusion angle, $\theta_{exc1}$, assumes the effect is neglected entirely (corresponding to Figure 3). The second exclusion angle, $\theta_{exc2}$, assumes the spacecraft is known to be somewhere in Earth orbit (corresponding to Figure 4). The exclusion angles required to completely ignore the effect of gravitational light deflection, $\theta_{exc1}$, are clearly too large (essentially the entire celestial sphere for the Sun). Thus, any practical implementation of the StarNAV framework will almost certainly require some accounting of the gravitational deflection of light. In this particular example, simply accounting for the spacecraft being in Earth orbit creates reasonable exclusion angles for all bodies other than the Earth itself (where it’s noted that Sun exclusion angles on the order of 10–20 deg are common for optical sensors). The deflection of light by Earth’s gravity is measurably affected by the spacecraft’s changing Earth-relative position and must be estimated as part of the StarNAV framework.

#### 3.2.2. Stellar Aberration

Stellar aberration—defined here as the change in apparent direction to a star due to the relative motion between the observer and the frame in which the reference star direction is defined—is a direct consequence of the relativistic addition of velocities. Although modern mathematical descriptions of stellar aberration make use of relativity, the existence of this effect predates Einstein by some time. The classical (Galilean) explanation is due to the landmark work of James Bradley in the early eighteenth century [103]. It was the tension between Bradley’s explanation of stellar aberration with the prevailing theories of light in the late nineteenth century that provided one of the principal motivations for Einstein’s development of the Special Theory of Relativity [104,105] (see Appendix A).

While the classical approach of Bradley is still used today—as, unfortunately, is the case for earlier work exploring stellar aberration for navigation [9]—the result is only correct to first order in v/c. As will soon become apparent, navigation by stellar aberration requires consideration of at least second (if not third) order terms in v/c, thus, rendering the classical approach ineffectual for the task at hand.

##### Effect of Stellar Aberration on Observed Direction to a Single Star

Stellar aberration is most straightforwardly explained using a Lorentz transformation to relate how a ray of light is seen by a stationary observer (e.g., zero velocity relative to the star catalog frame) and a moving observer (e.g., a telescope on the surface of Earth or a spacecraft).

Proceed, therefore, by introducing the common convention

(38) β=v/c

such that the Lorentz factor becomes

(39) $$\gamma =1/\sqrt{1-v^{T}v/c^{2}}=1/\sqrt{1-\beta^{T}\beta }$$

Through direct application of the Lorentz transformation (see Appendix A), the apparent direction $u_{i}^{\prime \prime }$ to the *i*-th star as measured by an observer aboard the moving spacecraft (light ray with tangent velocity $-cu_{i}^{\prime \prime }$) is related to the direction $u_{i}^{\prime }$ (see Equation (29)) to the same star as seen by a non-moving observer at same location (light ray with tangent velocity $-cu_{i}^{\prime }$) according to [10,59]

(40) $$u_{i}^{\prime \prime }=\frac{1}{\gamma \;(1+v^{T}u_{i}^{\prime }/c)}\left\{u_{i}^{\prime }+\left[\frac{\gamma }{c}+\left(\gamma -1\right)\frac{v^{T}u_{i}^{\prime }}{v^{T}v}\right]v\right\}$$

which after straightforward algebraic manipulation becomes

(41) $$u_{i}^{\prime \prime }=\frac{1}{\gamma \;(1+\beta^{T}u_{i}^{\prime })}\left\{u_{i}^{\prime }+\left[\gamma +\left(\gamma -1\right)\frac{\beta^{T}u_{i}^{\prime }}{\beta^{T}\beta }\right]\beta \right\}$$

(42) $$u_{i}^{\prime \prime }=\frac{1}{1+\beta^{T}u_{i}^{\prime }}\left[u_{i}^{\prime }+\beta -\left(\frac{1-\gamma }{\gamma }\right)\langle \beta \rangle \times \left(\langle \beta \rangle \times u_{i}^{\prime }\right)\right]$$

The equivalence of these expressions with the original result of Einstein for the aberration of light is shown at the end of Appendix A.

By employing the machinery of special relativity, the result of Equations (40)–(42) assumes locally flat spacetime. That is, the velocity v is the velocity of the spacecraft as seen by a stationary fictitious observer sitting at BCRF position r. Care must be taken in relating v to the spacecraft velocity as seen by observers at other locations or in different reference frames. The difference between the various relativistic representations of the spacecraft velocity with their Newtonian counterparts is generally $O(c^{-2})$, the implications of which are discussed in Section 5.

Therefore, proceeding undeterred, observe that the magnitude of β is generally small (e.g., $∥\beta ∥\leq 10^{-4}$ for objects in Earth orbit). Consequently, it becomes insightful to consider an expansion of Equation (42) about $\beta =0_{3\times 1}$,

(43) $$\begin{array}{cc} u_{i}^{\prime \prime }= & u_{i}^{\prime }\\ \; & +\left[u_{i}^{\prime }\times \left(\beta \times u_{i}^{\prime }\right)\right]\\ \; & -\left[\left({u^{\prime }}_{i}^{T}\beta \right)u_{i}^{\prime }\times \left(\beta \times u_{i}^{\prime }\right)+\left(1/2\right)\beta \times \left(u_{i}^{\prime }\times \beta \right)\right]\\ \; & +\left[{\left({u^{\prime }}_{i}^{T}\beta \right)}^{2}u_{i}^{\prime }\times \left(\beta \times u_{i}^{\prime }\right)+\left({u^{\prime }}_{i}^{T}\beta /2\right)\beta \times \left(u_{i}^{\prime }\times \beta \right)\right]\\ \; & +O(∥\beta {∥}^{4}) \end{array}$$

This expansion is equivalent to that reported in [10] and also the same to first order in β as [9,59] (these latter two only went to first order).

For a spacecraft with ∥v∥=38 km/s, the term linear in β is up to 26.1 arcsec, the term quadratic in β is up to 1.7 mas, and the cubic term in β is up to 0.1 μas. Given that the assumed sensor error is on the order of 0.1–1 mas, it is clear that one must consider terms up to second order in β. Furthermore, the linear term in β dictates the magnitude of the relationship between a perturbation in the velocity and the corresponding perturbation in the observed star LOS direction. The maximum sensitivity geometry leads to δϕ∼δv/c, such that 0.1 mas of measurement error in the star LOS direction would correspond to as much as 0.15 m/s of velocity error.

##### Effect of Stellar Aberration on Inter-Star Angle

The single-star stellar aberration model from Equation (42) may be used to develop a closed-form solution for the effect of stellar aberration on the angle between two stars. Therefore, after defining the observed angle between two stars as $\theta_{ij}^{\prime \prime }$,

(44) $${u_{i}^{\prime \prime }}^{T}u_{j}^{\prime \prime }=\cos\theta_{ij}^{\prime \prime }$$

substitution of Equation (42) and some algebraic manipulation leads to [10]

(45) $$\cos\theta_{ij}^{\prime \prime }={u_{i}^{\prime \prime }}^{T}u_{j}^{\prime \prime }=1-\left(1-{u_{i}^{\prime }}^{T}u_{j}^{\prime }\right)\left[\frac{1-\beta^{T}\beta }{\left(1+\beta^{T}u_{i}^{\prime }\right)\left(1+\beta^{T}u_{j}^{\prime }\right)}\right]$$

As with the single star case, this result may also be expanded about $\beta =0_{3\times 1}$. Recognizing that

(46) $${\left(1+\beta^{T}u_{i}^{\prime }\right)}^{-1}=1-\beta^{T}u_{i}^{\prime }+{\left(\beta^{T}u_{i}^{\prime }\right)}^{2}-{\left(\beta^{T}u_{i}^{\prime }\right)}^{3}+{O(∥\beta ∥}^{4})$$

simple substitution and grouping of like terms will show that

(47) $$\begin{array}{cc} \cos\theta_{ij}^{\prime \prime }={u_{i}^{\prime \prime }}^{T}u_{j}^{\prime \prime }= & {u_{i}^{\prime }}^{T}u_{j}^{\prime }+\left(1-{u_{i}^{\prime }}^{T}u_{j}^{\prime }\right)\\ \; & \times \left\{\left[\beta^{T}u_{i}^{\prime }+\beta^{T}u_{j}^{\prime }\right]\right.\\ \; & \;-\left[{\left(\beta^{T}u_{i}^{\prime }\right)}^{2}+{\left(\beta^{T}u_{j}^{\prime }\right)}^{2}+\left(\beta^{T}u_{i}^{\prime }\right)\left(\beta^{T}u_{j}^{\prime }\right)-\beta^{T}\beta \right]\\ \; & \;\left.+\left[\left(\beta^{T}u_{i}^{\prime }+\beta^{T}u_{j}^{\prime }\right)\left({\left(\beta^{T}u_{i}^{\prime }\right)}^{2}+{\left(\beta^{T}u_{j}^{\prime }\right)}^{2}-\beta^{T}\beta \right)\right]\right\} \end{array}$$

(48) $$\begin{array}{cc} \; & +O(∥\beta {∥}^{4}) \end{array}$$

This expression is equivalent to that reported in [10] and also the same to first order in β as [9] (the latter one only went to first order).

Assuming the LOS directions to the two stars are nearly perpendicular to one another ($u_{i}^{T}u_{j}\approx 0$) and a spacecraft with ∥v∥=38 km/s, the term linear in β is up to 37 arcsec, the term quadratic in β is up to 3.3 mas, and the cubic term in β is up to 0.2 μas. As with the single star case, sensor error on the order of 0.1–1 mas requires the consideration of the term that is second order in β.

### 3.3. Perturbations in Frequency of Stellar Spectra

#### 3.3.1. Gravitational Blueshift/Redshift

As a consequence of general relativity, starlight originating from outside our Solar System experiences a blueshift (increases in frequency) due to the potential field of the Sun and planets. Conversely, light from the Sun experiences a redshift (decrease in frequency) as it emanates outward through the Solar System. Following the same notational convention as used in Section 3.1.2, let $f_{i}$ be the frequency of light from the *i*-th star in the absence of (or infinitely far from) the gravitating bodies. Furthermore, let $f_{i}^{\prime }$ be the frequency of light from the *i*-th star as seen by a fictitious observer at BCRF position r with zero velocity. With these definitions, and considering only the spherically symmetric portions of the gravity field, general relativity suggests that (again, assuming the source to be infinitely far away) [106]

(49) $$\frac{f_{i}}{f_{i}^{\prime }}=z^{\prime }+1=1-\frac{1}{c^{2}}U\left(r\right)+O\left(c^{-4}\right)$$

where $z^{\prime }$ is from Equation (13) and U(r) is the local gravitational potential,

(50) $$U\left(r\right)\approx \sum_{B}\frac{GM_{B}}{∥r-r_{B}∥}$$

and, as before, *G* is the universal gravitational constant, $M_{B}$ is the mass of celestial body *B*, and $r_{B}$ is the BCRF position of celestial body *B*.

In most cases, this effect is dominated by the gravitational attraction from the Sun. For a spacecraft at an altitude of 410 km above the Earth’s surface, $z^{\prime }=-9.9\;\times \;10^{-9}$ (equating to an apparent velocity perturbation of $cz^{\prime }\;=\;2.96$ m/s) considering just the Sun. When both the Earth and Sun are considered, the blueshift increases to $z^{\prime }\;=\;-10.5\;\times \;10^{-9}$ (equating to an apparent velocity perturbation of $cz^{\prime }\;=\;3.16$ m/s).

#### 3.3.2. Relativistic Doppler Effect

The Doppler effect is another consequence of the Special Theory of Relativity. Let the frequency of light seen by a fictitious stationary observer at BCRF position r be given by $f_{i}^{\prime }$ (which one may obtain from Equation (49)), and let the frequency of light as seen by an observer aboard the moving spacecraft be given by $f_{i}^{\prime \prime }$. Again, using the same convention as for stellar aberration, denote the spacecraft velocity as seen by fictitious stationary observer as v. Assuming the light is emanating from the *i*-th star, the apparent direction to the star is $u_{i}^{\prime }$ for a fictitious stationary observer and $u_{i}^{\prime \prime }$ for the spacecraft. Define $n_{i}$ to be the tangent vector to the ray of light at r, such that

(51) $$u_{i}^{\prime }=-n_{i}^{\prime }$$

(52) $$u_{i}^{\prime \prime }=-n_{i}^{\prime \prime }$$

For convenience in comparing with common convention, define $\phi_{i}$ to be the angle between the ray of light’s tangent direction and the velocity vector,

(53) $$\cos\left(\phi_{i}^{\prime }\right)={\langle v\rangle }^{T}n_{i}^{\prime }=-{\langle v\rangle }^{T}u_{i}^{\prime }$$

(54) $$\cos\left(\phi_{i}^{\prime \prime }\right)={\langle v\rangle }^{T}n_{i}^{\prime \prime }=-{\langle v\rangle }^{T}u_{i}^{\prime \prime }$$

In his original 1905 paper introducing what would become known as the Special Theory of Relativity [104], Einstein presented the following expression for the relation between the frequency of light seen by observers in two different frames (the so-called relativistic Doppler effect)

(55) $$f_{i}^{\prime \prime }=\gamma \left(1-∥\beta ∥\cos(\phi_{i}^{\prime })\right)f_{i}^{\prime }=\gamma \left(1+\beta^{T}u_{i}^{\prime }\right)f_{i}^{\prime }$$

where γ is the Lorentz factor from Equation (39). Note that this is expressed in terms of the star direction as seen by the stationary fictitious observer, $u_{i}^{\prime }$. It is also possible to write the relativistic Doppler effect in terms of the star direction as seen by the moving observer, $u_{i}^{\prime \prime }$,

(56) $$f_{i}^{\prime \prime }=\frac{f_{i}^{\prime }}{\gamma \left(1+∥\beta ∥\cos(\phi_{i}^{\prime \prime })\right)}=\frac{f_{i}^{\prime }}{\gamma \left(1-\beta^{T}u_{i}^{\prime \prime }\right)}$$

which is found by the substituting the following expression for stellar aberration (see Appendix A) into Equation (55)

(57) $$\cos\left(\phi_{i}^{\prime \prime }\right)=\frac{\cos(\phi_{i}^{\prime })-∥\beta ∥}{1-∥\beta ∥\cos(\phi_{i}^{\prime })}$$

It is emphasized that Equations (55) and (56) are equivalent expressions for the relativistic Doppler effect, simply written in terms of the star direction as seen by different observers.

Within the context of StarNAV-DE navigation, the aberrated star direction direction $u_{i}^{\prime \prime }$ is likely not known with sufficient precision since the velocity is unknown. Therefore, it is generally better to employ Equation (55), as $u_{i}^{\prime }$ is more likely to be known in practice to the necessary precision.

As with stellar aberration, it is insightful to expand Equation (55) about $\beta =0_{3\times 1}$. Specifically, rearrange Equation (55) to find

(58) $$z_{i}^{\prime \prime }+1=\frac{f^{\prime }}{f^{\prime \prime }}=\frac{1}{\gamma \left(1+\beta^{T}u_{i}^{\prime }\right)}$$

The terms on the right-hand side may be individually expanded to $O(∥\beta {∥}^{3})$

(59) $$\gamma^{-1}=1-\frac{1}{2}\beta^{T}\beta +{O(∥\beta ∥}^{4})$$

(60) $${\left(1+\beta^{T}u_{i}^{\prime }\right)}^{-1}=1-\beta^{T}u_{i}^{\prime }+{\left(\beta^{T}u_{i}^{\prime }\right)}^{2}-{\left(\beta^{T}u_{i}^{\prime }\right)}^{3}+{O(∥\beta ∥}^{4})$$

Substitution of these results into Equation (58), grouping like powers of β, and neglecting terms of $O(∥\beta {∥}^{4})$ and higher,

(61) $$\begin{array}{c} z_{i}^{\prime \prime }=-\beta^{T}u_{i}^{\prime }+\left[{\left(\beta^{T}u_{i}^{\prime }\right)}^{2}-\frac{1}{2}\beta^{T}\beta \right]-\left(\beta^{T}u_{i}^{\prime }\right)\left[{\left(\beta^{T}u_{i}^{\prime }\right)}^{2}-\frac{1}{2}\beta^{T}\beta \right]+{O(∥\beta ∥}^{4}) \end{array}$$

which agrees with more complicated expansions of the relativistic Doppler effect (e.g., [106,107]) when appropriate assumptions are made regarding the source and observer. Note, of course, that if one only retains the linear term in β the result is the classical (non-relativistic) Doppler effect

(62) $$\begin{array}{c} z_{i}^{\prime \prime }=-\frac{1}{c}v^{T}u_{i}^{\prime }+{O(∥\beta ∥}^{2}) \end{array}$$

Returning to the $O(∥\beta {∥}^{4})$ expansion from Equation (61) and assuming a spacecraft with ∥v∥=38 km/s, the term linear in β is up to $1.27\times 10^{-4}$, the term quadratic in β is up to $8.03\times 10^{-9}$, and the cubic term in β is up to $1.02\times 10^{-12}$. The worst-case geometry (when $u_{i}^{\prime \prime }$ and v are collinear) leads to $\delta z^{\prime \prime }∼\delta v/c$, such that the second-order term could contribute a velocity error as large as 2.4 m/s and the third-order term could contribute a velocity error as large as 0.3 mm/s.

It is clear, therefore, that second-order terms are likely required in the measurement model and that use of the non-relativistic (linear) Doppler effect is not generally appropriate. To avoid an unnecessary measurement bias, it is suggested that the nonlinear measurement model of Equation (55) be used in practice.

#### 3.3.3. Remarks on the Combination of Gravitational Blueshift and Relativistic Doppler Effect

The result of Equation (55) and the resulting expansion of Equation (61) consider only the effects of special relativity. It is worth briefly noting that these results be combined with Equation (49) to provide a compact expression for $f/f^{\prime \prime }$. Therefore, recalling Equation (16), the total frequency shift relative to the reference spectrum (zero potential at the SSB) is

(63) $$\frac{f}{f^{\prime \prime }}=\left(\frac{f}{f^{\prime }}\right)\left(\frac{f^{\prime }}{f^{\prime \prime }}\right)=\left(z^{\prime }+1\right)\left(z^{\prime \prime }+1\right)$$

Proceed by noting $z^{\prime }$ is given by Equation (49) and $z^{\prime \prime }$ by Equation (58). Therefore, substituting expansions for $z^{\prime }$ and $\gamma^{-1}$ only,

(64) $$\frac{f}{f^{\prime \prime }}=\left[1-\frac{1}{2c^{2}}v^{T}v-\frac{1}{c^{2}}U\left(r\right)\right]{\left(1+\frac{1}{c}v^{T}u_{i}^{\prime }\right)}^{-1}+O\left(c^{-4}\right)$$

which is the expression commonly seen for relating barycentric and observer spectra (e.g., [87]).

## 4. Preliminary Feasibility Assessment of StarNAV Measurements

This section considers the practical feasibility of obtaining StarNAV-SA and StarNAV-DE measurements. StarNAV-SA measurements are found to be achievable with existing technology, although challenges remain with regard to instrument size, inter-instrument alignment, and vibration isolation. StarNAV-DE measurements of suitable accuracy are not presently achievable due to difficulties with stellar spectra stability and instrument calibration. Details in support of these conclusions are now presented.

### 4.1. Feasibility of StarNAV-SA Measurements

The angular precision required to measure stellar aberration has been available for over a century. Indeed, state-of-the-art scientific instrumentation is presently capable of providing star bearing measurements with errors 2–3 orders of magnitude better than necessary for navigation with the StarNAV-SA technique.

The effect of stellar aberration occurs at all wavelengths of light and for every star. Consequently, the system designer is free to select guide stars from 100s of bright stars of suitable astrometric quality that are well-distributed throughout the celestial sphere. Furthermore, these stars may be observed in whatever wavelengths are most convenient (e.g., visible, infrared, X-ray). This is a substantial advantage of StarNAV-SA measurements when compared to XNAV, as the latter is limited to observing a relatively small set of stable millisecond pulsars (many of which are not very bright).

Although individual instrument components may measure directions to a particular star, the StarNAV-SA technique ultimately only uses inter-star angles for navigation. Since the inter-star angles seen by the sensor system are the same regardless its orientation, the choice of using only inter-star angles allows navigation without the need of an inertial attitude estimate better than the star sighting measurements themselves. This is of critical importance, as obtaining attitude estimates at the level of 0.1 mas is a daunting task—and likely impossible if the velocity is unknown *a priori*.

**Figure 5:**
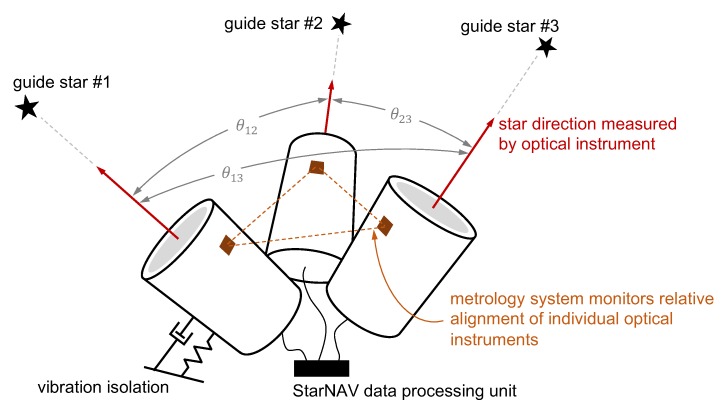
Notional diagram of a StarNAV-SA sensor system used to measure perturbations in inter-star angles, $\theta_{ij}$. A separate optical instrument (three depicted in this example) is used to observe each guide star. Orientation between the individual optical instruments is monitored by a metrology system. The entire StarNAV-SA sensor system requires vibration isolation as well as fine pointing (not illustrated) to keep guide stars within the instrument FOVs.

The present work presumes that a generic StarNAV system (see Figure 5) would need to measure the angle between two stars (separated from each other by a large angle) with an error on the order of 0.1–1 mas. Such bearing precision to a single star is possible with either a conventional direct imaging system (i.e., a telescope) or an interferometer. The primary challenge is the size of these systems and their compatibility with the constraints of a navigation instrument.

The large angle between stars likely requires that a separate optical instrument (either a telescope or an interferometer) be used to observe each star. Consequently, error in the inter-star angle is driven not only by the single-instrument error, but also by the error in the relative alignment of the instruments. Achieving long-term stability in instrument alignment at the 1 mas level or better is difficult in practice and a metrology system will likely be required to monitor relative alignment. Although such metrology systems are complicated, they have been proposed in the past for space systems of various sizes.

Obtaining bearing measurements with errors below 1 mas places considerable requirements on instrument pointing and vibration. Thus, all StarNAV systems are expected to require vibration isolation in practice. In some cases the StarNAV instrument platform may also require its own fine pointing system to obtain pointing control and slew rates that are be beyond the generic attitude control capability of the host spacecraft.

In summary, there are three primary considerations in assessing the practical feasibility of a StarNAV-SA system: (1) performance and size of a single optical instrument, (2) alignment between optical instruments, and (3) pointing and vibration. This is shown pictorially in Figure 5. The following subsections consider each of these areas in more detail and outline some of the major challenges in implementing such a system.

#### 4.1.1. Performance of Candidate StarNAV-SA Optical Instruments

There has been considerable past work on optical instruments capable of collecting the measurement type required for StarNAV. Of interesting historical note, a design for a “hyper-accuracy space sextant” was developed in the late 1960s as a derivative of the Apollo space sextant. This sensor was designed to measure inter-star angles for a theoretical crewed interstellar mission, thus, allowing for autonomous navigation by principles similar to those reported in this work. A summary of the optical design appears in [69] and a collection of detailed schematics appear in [108]. This sensor was capable of measuring inter-star angles with an error of around 0.5 arcsec (500 mas). While impressive for a completely manual system, the hyper-accuracy space sextant’s measurement error is still 2–3 orders of magnitude larger than needed for navigation by stellar aberration in most cases.

It is desirable (if not required) in most cases to have an automatic—instead of manual—system. Typical star trackers and other conventional camera-like navigation sensors are not generally diffraction limited. Instead, these sensors employ intentional defocus to spread the photons from a single star across multiple pixels, ultimately allowing star centroids to be found with an accuracy of about 0.1 pixel [54,109]. At the sensor system level, star trackers may provide individual star bearing errors on the order of 1 arcsec. A more specialized astrometric sensor is clearly required for the present application. Therefore, the following discussion briefly considers the efficacy of staring telescopes and interferometers for obtaining StarNAV-SA measurements. While other techniques exist, such as scanning systems [110], a broader instrumentation trade is left for later work.

The accuracy for both telescopes and interferometers is fundamentally limited by diffraction and photon noise, providing a performance floor for even a perfectly built sensor. This limit is straightforward to derive from first principles in many ways, such as the methods of Falconi [111] or Lindegren [112,113].

Diffraction occurs as starlight interacts with the edges of the aperture on a telescope or interferometer. This effect is generically described by the Fresnel-Kirchhoff diffraction integral [114,115]. In the case where the diffracted light is focused onto a detector with an optical system, this is well approximated by the Fraunhofer diffraction equation—which, for a circular aperture, produces the celebrated Airy pattern (intensity pattern on the focal plane for the best-focused point source of light) [112,115,116],

(65) $$I\left(\phi \right)=\frac{N}{\pi }{\left[\frac{J_{1}\left(kD\phi /2\right)}{\phi }\right]}^{2}$$

where $J_{1}$ is the Bessel function of the first kind of first order, *D* is the aperture diameter, ϕ is the angle from the true star center, *N* is the total number of photons, and k=2π/λ is the wave number. The Airy pattern dictates the resolution of an optical system with a circular aperture by defining the minimum angular separation required to distinguish two point sources from one another. This resolution limit (the so-called Rayleigh criterion [117]) is generally taken to be the first dark band in the Airy pattern, which occurs at [115]

(66) $$\phi_{DL}\approx 1.22\frac{\lambda }{D}$$

The same approach may be used to determine the diffraction limited resolution of an interferometer, [118]

(67) $$\phi_{DL}\approx \frac{\lambda }{2B}$$

where *B* is the interferometer’s baseline.

It is essential to recognize that the Rayleigh criterion represents the system’s resolution and not its accuracy. In many cases, the accuracy of a bearing measurement to a particular source may be many orders of magnitude better than $\phi_{DL}$.

Recognizing that the diffraction pattern is essentially the probability density function (PDF) for where a photon will strike the focal plane, one may attempt to find the maximum likelihood estimate (MLE) of the star direction by minimizing the negative log-likelihood function. Under such a scheme, one may show the Cramér-Rao bound for the variance to be [113]

(68) $$\sigma_{\phi }^{2}\geq \frac{\lambda^{2}}{16\pi^{2}\Delta x^{2}N}$$

where Δx is the root-mean-square (RMS) extent of the aperture in the *x*-direction. For a circular aperture with area $A=\pi D^{2}/4$ one may analytically compute Δx for a filled-aperture telescope

(69) $$\Delta x_{tel}^{2}=\frac{1}{A}\int_{0}^{2\pi }\int_{0}^{D/2}r^{2}\cos^{2}\left(\theta \right)r\;dr\;d\theta =\frac{D^{2}}{16}$$

or for an interferometer consisting of two apertures of area *A* separated by a baseline *B*

(70) $$\Delta x_{int}^{2}=\frac{1}{2A}\int_{0}^{2\pi }\int_{0}^{D/2}\left\{{\left[r\cos(\theta )-B/2\right]}^{2}+{\left[r\cos(\theta )+B/2\right]}^{2}\right\}r\;dr\;d\theta =\frac{B^{2}}{4}+\frac{D^{2}}{16}$$

and, assuming B≫D,

(71) $$\Delta x_{int}^{2}\approx \frac{B^{2}}{4}$$

Consequently, the Cramér-Rao bound for the accuracy of a diffraction limited telescope is

(72) $$\sigma_{\phi_{tel}}\geq \frac{\lambda }{\pi D\sqrt{N}}$$

or, for an interferometer,

(73) $$\sigma_{\phi_{int}}\geq \frac{\lambda }{2\pi B\sqrt{N}}$$

These expressions are identical to those of Falconi [111] and Lindegren [112,113], which may also be found elsewhere in slightly different forms (e.g., [119]). The total number of photons collected by a single aperture of area *A* may be computed as

(74) N=Anτ

where *n* is the photon flux of the observed star as measured by the detector (see Section 3.1.3) and τ is the exposure time. For an interferometer with two apertures (each with diameter *D*), one finds that

(75) $$N=\left\{\begin{array}{cc} \pi n\tau D^{2}/4 & \mathrm{filled}\text{-}\mathrm{aperture}\;\mathrm{telescope}\\ \pi n\tau D^{2}/2 & \mathrm{two}\text{-}\mathrm{aperture}\;\mathrm{interferometer} \end{array}\right.$$

In practice, the actual performance of a system will necessarily be worse than the lower bounds of Equations (72) and (73) due to stray light, detector noise, quantization error (pixelization) on a digital sensor, imperfections in construction of the optical system, and other real-world challenges. Different choices, especially in the numerical scheme used to compute the centroid of the star diffraction pattern (see [112] for a few different examples) cause the leading coefficient to change slightly when compared to Equations (72) and (73). These changes tend to be rather small in magnitude, and modern sensor systems can come very close to achieving the limiting accuracies.

**Figure 6:**
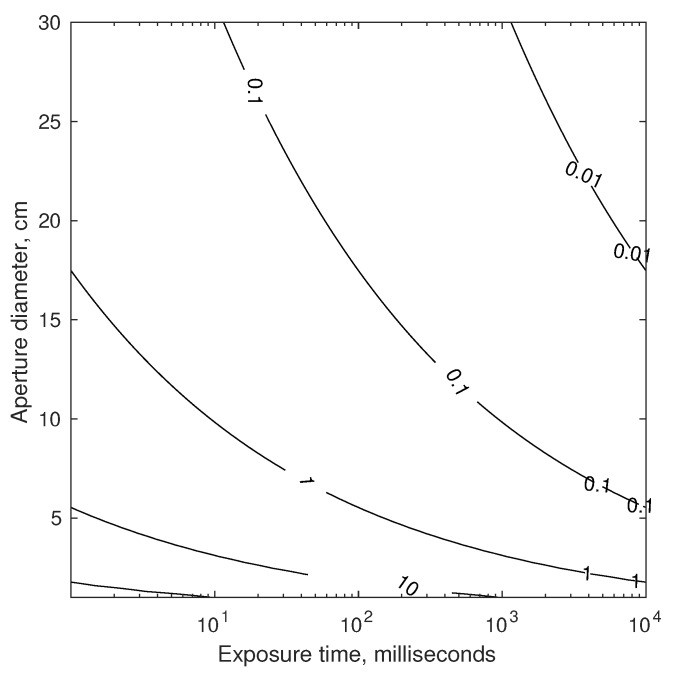
Contours of $\sigma_{\phi_{tel}}$ in milliarcseconds (mas) for a telescope viewing a star of magnitude $m_{V}=3$. Computed using Equation (72).

**Figure 7:**
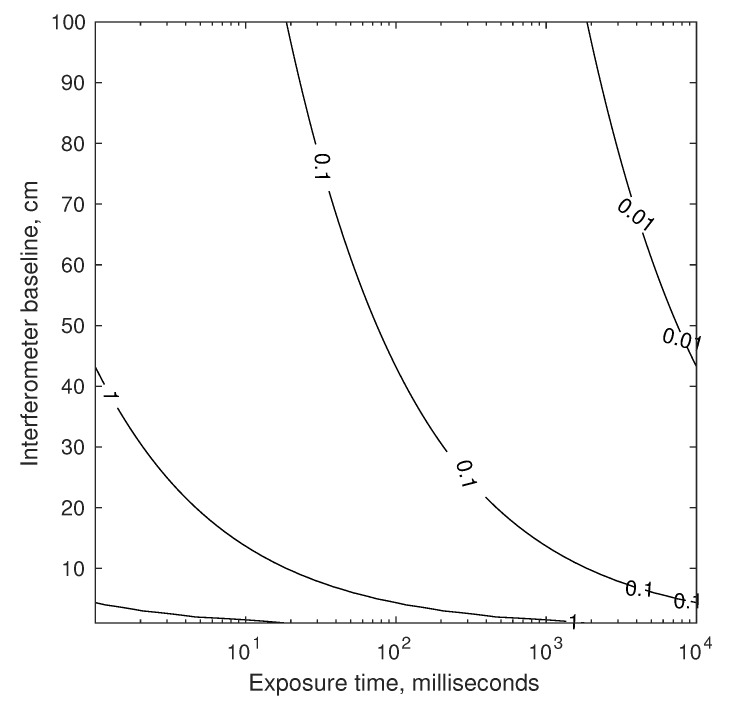
Contours of $\sigma_{\phi_{int}}$ in milliarcseconds (mas) for an interferometer with two 2.5 cm apertures viewing a star of magnitude $m_{V}=3$. Computed using Equation (73).

The theoretical results of Equations (72) and (73) may be used to compare the limiting accuracy of a telescope or interferometer. Assuming one views a star of magnitude $m_{V}=3$ with a CCD detector (measured flux of $n\approx 1.729\times 10^{9}$ photons/m${}^{2}$/second, see Table 1) near the middle of the visible spectrum (λ=550 nm), it is possible to evaluate telescope accuracy as a function of aperture diameter and exposure time (see Figure 6). For the interferometer, if each of the two apertures are 2.5 cm in diameter (chosen to keep them small for a navigation sensor), it is possible to evaluate interferometer accuracy as a function of baseline and exposure time (see Figure 7).

**Figure 8:**
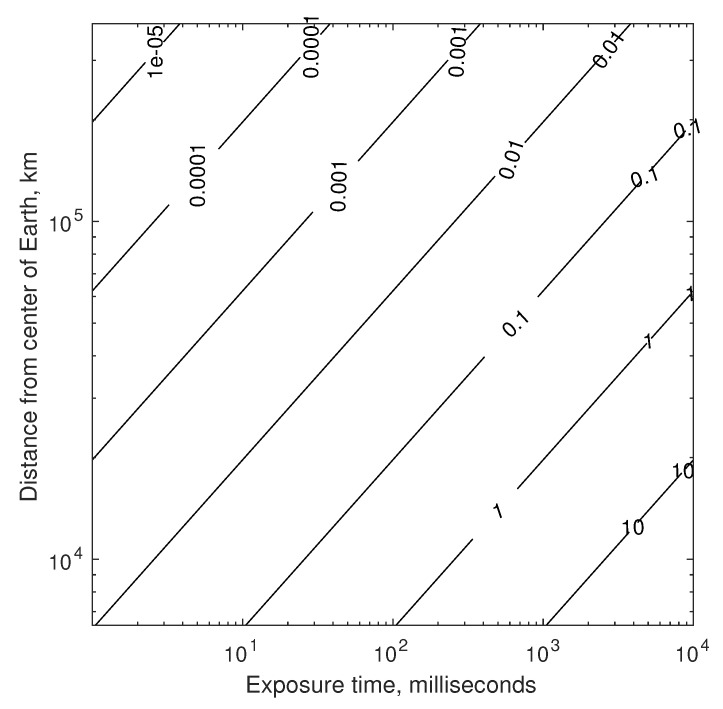
Contours of the change in inter-star angle change in milliarcseconds (mas) between the beginning and end of the specified exposure time for a spacecraft at varying distances from the Earth.

Temporarily setting aside the challenges of pointing and vibration (more on this in Section 4.1.3), a suitable exposure time is limited by the changing velocity (and, thus, changing inter-star angle $\theta_{ij}^{\prime \prime }$) over the time interval. For two stars with $\theta_{ij}=90$ deg, the worst-case change in apparent inter-star angle over the time τ for a spacecraft in Earth orbit is shown in Figure 8. Consequently, the maximum allowable exposure time is mission dependent.

Taken together, the results of Figure 6, Figure 7 and Figure 8 provide the rationale for assuming 0.1–1 mas as an achievable standard deviation for StarNAV-SA inter-star angle measurements. These results also suggest that substantially better inter-star angle measurements will be difficult to achieve using a telescope or interferometer if one wishes to keep the system’s size within reasonable values for a navigation instrument.

Which optical instrument (telescope, interferometer, or something else) is best will depend on application-specific needs. Beyond the differences apparent from a comparison of Figure 7 and Figure 8, there are numerous other important considerations that affect performance, mass, size, power, and cost. Some first-order considerations are now discussed for a StarNAV telescope and interferometer, with feasibility assessed both by analysis and by analogy. Substantial forward work exists to evaluate actual efficacy of each through the detailed engineering design of such a system.

##### Telescopes

A telescope is essentially a camera with a narrow FOV and (usually) a large aperture. The primary design considerations with using such a system to measure the bearing to stars at the 0.1–1 mas level are mostly related to system size (e.g., focal length, mass of lens/mirror assembly, light baffling) and calibration.

Achieving the angular accuracy of Equation (72) requires diffraction limited imaging, which occurs when the pixel pitch is much smaller than the size of the Airy disk to allow for many samples of the diffraction pattern within its first minimum. That is, using Equation (66), one requires that

(76) $$f_{\ell }\phi_{DL}=1.22\frac{f_{\ell }\lambda }{D}\gg \mu_{pix}$$

where $f_{\ell }$ is the effective focal length. If this is not the case, bearing error is driven more by focal plane quantization and is necessarily worse than the limit of Equation (72)

As an illustrative example, consider the Celestron Astro Fi 102mm Maksutov-Cassegrain telescope [120], having an optical tube with a diameter of 11.7 cm, a length of 38.1 cm, and a mass of 2.7 kg. This telescope has an aperture of D=10.2 cm and an effective focal length of $f_{\ell }=1.325$ m, thus producing an Airy disk of radius 8.7 μm for visible light at λ=550 nm. Assuming a pixel pitch of 1 μm, one would have an area of about 238 pixels inside the first minimum of the Airy disk. If the centroid is found to within 0.01 pixel (a reasonable goal for diffraction limited astrometry) by fitting the observed diffraction pattern with a model in a MLE sense, then this results in a bearing error of around 1.6 mas. Conversely, using Equation (72) and assuming a star of $m_{V}=3$ is observed with an exposure time of 5 ms, one would obtain an ideal diffraction limited accuracy of $\sigma_{\phi_{tel}}\approx 1.3$ mas. Using the relations from Section 3.2.2.1, a bearing error of 1.3 mas to a single star corresponds to a velocity error of around 1.9 m/s. The objective of this example is not to suggest this particular telescope be used for Star-NAV, but to illustrate that reasonable performance may be achieved with commercial-off-the-shelf (COTS) optics available to amateur astronomers. Superior performance and packaging may certainly be achieved for a purpose-built spaceflight instrument.

Beyond the optics themselves, successful imaging of stars in the space environment usually requires light baffling to block stray light. This is essential to maintain the high signal-to-noise ratio (SNR) assumed in the analysis so far. Light baffles can be quite large, especially for telescopes with large apertures and narrow FOVs, and are expected to be of significant concern in the design of a StarNAV telescope. For a narrow FOV telescope where the stray light exclusion angle is much larger than the FOV ($\phi_{ex}\gg \phi_{FOV}$) one may estimate the length $\ell_{b}$ of a simple baffle according to

(77) $$\ell_{b}\approx D/\tan\left(\phi_{ex}\right)$$

Therefore, for the example telescope in the preceding paragraph with D=10.2 cm, a light baffle blocking stray light originating from beyond 30 deg of the telescope’s boresight would be about 18 cm in length. More detailed mathematical models for light baffle design may be found in [121].

##### Interferometers

The fundamental advantage of interferometry is that one can replace a large monolithic telescope with two small apertures separated by a large baseline. When very high accuracy is needed it is generally easier to increase the baseline than to increase the size of a large single aperture. The accuracy advantages of the interferometer, however, come at the expense of increased complexity.

Numerous interferometer systems for space-based astrometry have been proposed, many with accuracies approaching 1 μas [122,123,124,125]. Most of the past interferometer system designs are one of two fundamental types: (1) a Michelson stellar interferometer [126,127] or (2) a Fizeau interferometer. While each type of interferometer has its own advantages and disadvantages, the particular embodiment of such a system is not important for the present high-level feasibility assessment.

**Figure 9:**
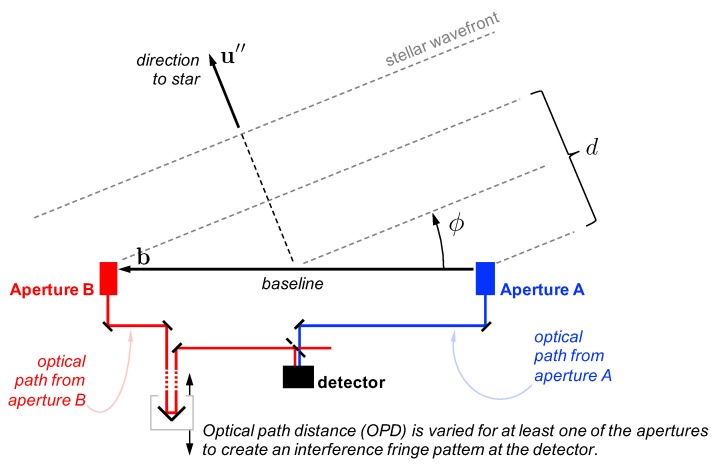
Notional schematic of the geometry for a 2D interferometer with two apertures.

An interferometer can be used to find the direction to a star by measuring the optical path delay (OPD) necessary to create interference fringes between starlight collected at two apertures separated by a baseline b (where ∥b∥=B). To motivate the mathematical development, consider the notional 2D interferometer in Figure 9. It is clear from geometry that the OPD may be computed as

(78) $$d=b^{T}u^{\prime \prime }+\kappa$$

where *d* is the OPD, b is the baseline vector from one aperture to the other, $u^{\prime \prime }$ is the observed star direction (see Section 3.2.2), and κ is the instrument’s OPD bias. Thus, defining the angle between b and $u^{\prime \prime }$ to be (π/2−ϕ) as shown in Figure 9, one may find ϕ according to

(79) $$\phi \approx \sin\left(\phi \right)=\cos\left(\pi /2-\phi \right)=\frac{b^{T}u^{\prime \prime }}{B}=\frac{d-\kappa }{B}$$

Consequently, if one has an error in the OPD measurement of $\sigma_{d}$, an error in the OPD bias of $\sigma_{\kappa }$, and an error in the baseline length of $\sigma_{B}$, then the error in the measured angle is

(80) $$\sigma_{\phi_{OPD}}^{2}=\frac{1}{B^{2}}\left[\sigma_{d}^{2}+\sigma_{\kappa }^{2}+\frac{{\left(d-\kappa \right)}^{2}}{B^{2}}\sigma_{B}^{2}\right]$$

To achieve the theoretical limit of Equation (73) it is required that $\sigma_{\phi_{OPD}}\;<\;\sigma_{\phi_{int}}$, otherwise the system’s accuracy would be driven by the ability to measure the OPD and not by the diffraction limit. Contemporary commercial interferometers are capable of measuring the OPD with accuracy at the nanometer level, while specialized space-based interferometers for science applications are now able to measure OPD at the picometer level [128,129]. OPD control at the nanometer level is also possible on a spacecraft [130,131]. Picometer sensing and nanometer control appears to be representative of the current state-of-the-art for space-based interferometry, although future technological advances are likely to result in improved performance. Regardless of the performance, the complexity of current OPD sensing and control systems represent one of the major disadvantages of interferometers when compared to telescopes.

As an illustrative example, consider an interferometer with two 2.5 cm apertures separated by a 30 cm baseline. Assuming one views a $m_{V}=3$ star in the visible spectrum (e.g., λ=550 nm) with an exposure time of 5 ms, the diffraction limited accuracy from Equation (73) is $\sigma_{\phi_{int}}=0.65$ mas. If one is capable of measuring the OPD to within 1 nm, this would correspond to an angle accuracy of $\sigma_{\phi_{OPD}}\approx 0.69$ mas. In principle, therefore, one should be able to achieve 0.1–1 mas accuracy using an interferometer with a 30 cm baseline. This conclusion is further supported by the optical design of the Newcomb mission, which was a proposed low-cost interferometry mission having a stack of 3–4 Michelson stellar interferometers (each with a 30 cm baseline) to obtain star directions on the order of 0.1 mas [132,133].

Finally, one of the few—if not only—documented examples of an optical instrument specifically designed for navigation by stellar aberration is an interferometer due to researchers at the U.S. Naval Observatory, with a brief discussion appearing in [9]. This instrument is described as being 88×90×26 cm in size and capable of measuring a star’s direction to within about 20 μas (substantially better than the 0.1–1 mas suggested in this work). There appear to be few references to this system outside of [9] and it is unclear if a prototype was ever built.

#### 4.1.2. Instrument Alignment and Metrology

The StarNAV-SA measurement type requires that individual stars be separated by a large angle, resulting in the need for a separate optical instrument to observe each star. In a real sensor system, even after great care has been taken to athermalize individual optical instruments [134], thermal strain (and other real-world effects) can easily alter the relative alignment between the separate optical instruments by many arcseconds. While careful system design and material selection may help, it is unlikely that relative alignment will stay truly fixed at the milliarcsecond level as the vehicle changes orientation relative to the Sun. Indeed, optical bench designs for past space missions [135,136] suggest that maintaining instrument alignment below the arcsecond level by passive means alone would be prohibitively difficult with conventional designs and materials. Therefore—since these thermal deformations cannot be eliminated, are difficult to model, and are not predictable in real-time with the requisite precision—the common solution is to have a metrology system to measure changes in the alignment between the various instruments. There are many different designs for such metrology systems, and these have been demonstrated with some success for both ground-based [137,138,139] and space-based [140,141,142] wide-angle astrometry. It is expected that a metrology system will be required to monitor component alignment in any future StarNAV-SA sensor system.

Furthermore, the analysis in Section 7 shows that the precision is more important than accuracy in the inter-star angle measurements. The measurement bias between a particular star pair (which comes from a combination of star catalog error and misalignment between the instrument components sighting each star in the pair) may be estimated so long as it changes very slowly with respect to the vehicle dynamics. Thus the stability of the alignment between the StarNAV instrument components is likely to be of paramount importance.

#### 4.1.3. Instrument Platform Pointing and Vibration

As a navigation sensor, it is not desirable to pass the pointing and vibration requirements of the StarNAV optical instruments on to the host spacecraft. This may be partially avoided by developing a fine-pointing system and a vibration isolation system. Although sometimes complicated and expensive, technologies for both are now briefly reviewed. The detailed consideration of these issues is deferred to later work.

The collection of StarNAV-SA measurements at the 0.1–1 mas level will require pointing of the optical instruments to at least 1 arcsec (perhaps even less, depending on specifics of instrument design). Fine-pointing systems with noise equivalent angle performance at this level have been developed for numerous spacecraft scientific payloads of all sizes (from CubeSats to large space telescopes) [143,144,145,146,147].

Vibrations are expected from various sources, with the dominant source on robotic spacecraft often due to host spacecraft’s reaction wheel assembly (RWA) [148]. Crewed vehicles, such as the ISS [149], often have a less favorable vibration environment due to movement of the human crew about the cabin and other crew-related systems (e.g., air circulation, gas venting). Regardless of the source, vibration isolation systems are common feature of scientific optical payloads of all sizes [150,151,152].

### 4.2. Feasibility of StarNAV-DE Measurements

Although the Doppler shift of starlight (or sunlight) may seem to be the most obvious means of autonomous velocity estimation, it is shown here to be a poor approach for many navigation applications within the Solar System. This finding is important to record in detail despite the negative result, as using the Doppler effect for stellar (or solar) or autonomous navigation has been repeatedly suggested over the last sixty years [4,5,60,61,62,63]. With the exception of [8,62], few authors seem to fully appreciate the practical difficulties associated with this approach.

There are three reasons that navigation by stellar (or solar) spectral shift is difficult—all of which must be addressed to make such a system worth implementing in practice. The first challenge is poor stability of the stellar spectra over both short and long timescales, making stellar spectra an unreliable signal for navigation. The second challenge is the need for a frequency calibration source of suitable stability and accuracy. The third challenge is measuring the spectral shift with the necessary accuracy within the context of an autonomous navigation system. The first may be a fatal flaw, the second makes the approach less desirable, and the third is likely solvable in the future with improved technology. Each of these challenges is now discussed.

#### 4.2.1. Stability of Stellar Spectra for Radial Velocity Estimation

The autonomous estimation of a spacecraft’s velocity relative to the SSB directly from the observation of stellar spectra requires comparison with reference spectra located at the SSB. Such reference spectra are presumably computed as discussed in Section 3.1.2, where a particular star’s spectrum ($f_{*}$) is transferred to the SSB at zero potential (*f*) according to

(81) $$f_{*}=\left(1+z_{B}\right)f$$

As should be evident by this point, the frequency shift $z_{B}$ is not constant and its stability is a significant concern in the practical efficacy of StarNAV-DE measurements. Indeed, the mean value of $z_{B}$ varies considerably on the timescale of a few minutes and the accuracy with which $z_{B}$ can be estimated varies with the level of the star’s activity. Consequently, the apparent radial velocity to a star is not simply the projection of the mean relative velocity (i.e., the difference between the spacecraft’s BCRF velocity and the star’s velocity obtained from a star catalog; e.g., [153]) onto the direction $u_{i}$.

The nearly constant radial velocity between the SSB and the star’s barycenter is corrupted by numerous effects, with the most important being (1) oscillations of the star’s surface due to acoustic waves, (2) motion of granules on the star’s surface, (3) interplay of star rotation with effects from surface activity, and (4) the motion of the star about the barycenter of its system. All of these effects contribute to perturbations on the order of a 10s of cm/s to a few m/s in the disk-integrated radial velocity.

Acoustic waves cause oscillations in the surface of a star. Depending on the star (type and evolutionary stage), disk-integrated radial velocity perturbations from *p*-mode oscillations can range from around 10 cm/s to 4 m/s with timescales of only a few minutes [154]. For example, Sun-like stars typically have mean *p*-mode amplitudes on the order of 10–60 cm/s (18.7 cm/s for the Sun) with peak power around 1–5 mHz (periods of 3.3–16.7 min; period of 5.5 min for the Sun) [155]. Local velocities on the star’s surface can be much higher, but these are due to modes of high harmonic degree that largely vanish in disk-integrated measurements.

Granulation describes localized convection patterns on the stellar surface, which often have lifetimes on the order of a few minutes to many hours. Individual convection patterns can have vertical velocities on the order of 100 s of m/s [156]. However, since millions of these are viewed simultaneously in a disk-integrated measurement, their collective contribution to disk-integrated radial velocity measurements is generally a few m/s or less. While individual granules have a relatively short lifetime, the overall effect of granulation on radial velocity changes somewhat in amplitude with the level of stellar activity (having a timeline of years; e.g., 11-year cycle for the Sun). This occurs because increased magnetic activity on the star’s surface can locally limit the size of the granules [157], thus, decreasing homogeneity in granule behavior across the observed stellar disk.

Although one might not expect stellar rotation to contribute much to the disk-integrated radial velocity (ideally, the velocity of the hemisphere moving towards the observer is canceled out by the velocity of the hemisphere moving away from the observer), interaction of the star’s rotation with surface activity phenomena does cause systematic perturbations in the measured radial velocity. This happens in a few ways. First, spectral line asymmetries induced by granulation are enhanced by the star’s rotation [158], thus, reducing the accuracy in computing the shift between the reference and observed spectra. Second, the rotational symmetry of the stellar disk is broken by spots and plages—thus, causing one hemisphere to contribute more than the other to radial velocity as these defects move across the observed stellar disk [159,160]. Third, most stars do not truly rotate as a rigid body [161], which further breaks the rotational symmetry across the observed stellar disk. All together, these effects can contribute perturbations in radial velocity on the order of 1–100 m/s with timescales of many days (depends on the rotational period of the star). From a navigation standpoint, it is likely that one can judiciously choose less active stars to keep the radial velocity errors from this source on the order of only a few m/s.

Finally, there are long-term oscillations in a star’s radial velocity due to the star’s motion about the barycenter of its system induced by the gravitational attraction of its planets. Indeed, the scientific community has had great success in using these oscillations in radial velocity to compute the size and orbit of planets—making this a powerful tool in the search for planets in a star’s habitable zone [162]. Although some have suggested the oscillation in radial velocity from exoplanets could be used for navigation (e.g., [63]), this does not seem plausible as the amplitude of such oscillations is too low (a few cm/s) and the period is too long (months to years). From the standpoint of autonomous navigation, where the timescale of obtaining a velocity solution must be on the order of seconds to minutes (depending on the spacecraft orbit), the periods of stellar oscillations from exoplanets are long enough that they are more appropriately handled as a bias in the navigation filter.

Of the effects discussed here, stellar oscillation and granulation are especially problematic for autonomous navigation because of difficulties in predicting their contribution to radial velocity and their timescales of a few minutes to a few hours. The conventional technique for removing these effects for science observations is to collect measurements with exposure times of many minutes to average out stellar oscillation [163] and at multiple times throughout a night (for Earth-based observations) to average out granulation [154]. Although multiple minutes-long exposures separated by many hours may be acceptable for navigation in heliocentric orbits far from the Sun (e.g., final portions of interplanetary cruise to outer planets, Kuiper belt tour) or in interstellar flight, the velocity changes too quickly for such exposure times to be useful in Earth orbit or for the majority of contemporary exploration missions. A different technique is likely necessary to account for these effects in an autonomous navigation system.

Another way to remove the radial velocity perturbations from stellar oscillation and granulation is to compare the same stellar spectrum as seen by two observers at two different locations. Since the spectrum is perturbed by the same amount at both locations (accounting for the light-time delay), these perturbations are unimportant if one only cares about the relative velocity between the two observers. This fundamental idea may be applied in at least two straightforward ways. First, the spectrum simultaneously observed on two different spacecraft communicating with one another could be compared to determine the relative velocity (in the direction of the star) between the two. While this could contribute to autonomous relative navigation [64], it does little to advance the need for autonomous absolute navigation. Second, using Sun instead of stars, the solar spectrum measured directly by the spacecraft could be compared with the spectrum reflected off a nearby celestial body with a well-known ephemeris. Although this has been proposed in the past [65,66], additional work is required to determine the true efficacy of such an approach.

#### 4.2.2. Suitable Source for Frequency Calibration

The Doppler shift of stellar (or solar) spectra may be measured in a number of ways, such as through the resonant scattering technique (e.g., the BiSON network that monitored shifts in the solar potassium Fraunhofer line [164], the Global Oscillations at Low Frequency (GOLF) experiment on SOHO that monitored shifts in the solar sodium Fraunhofer lines [165]), through diffraction gratings (e.g., Gaia Radial Velocity Spectrometer (RVS) [166]), High Accuracy Radial Velocity Planet Searcher (HARPS) [167]), or through other techniques (e.g., [168]).

The majority of modern systems claiming accuracy in radial velocity below 10 m/s make use of a spectrograph [162,169]. Fundamentally, sensors of this class disperse incoming starlight by its wavelength and capture the resulting spectra with a detector array (e.g., CCD, CMOS). This necessarily requires a frequency source to calibrate the observed spectra against a known absolute reference. While such calibration may be done using a reference emission lamp (where well-known emission lines are observed concurrently with the stellar spectra; typically a ThAr lamp [167,170]) or passing the starlight through a gas absorption cell (this imprints well-known absorption lines onto the stellar spectra; typically an iodine absorption cell [171,172]), these conventional approaches generally limit radial velocity measurement accuracy to a few m/s. Furthermore, while it may be possible to use ThAr emission lamps to achieve accuracy on the order of 10s of cm/s [167], such emission lamps are likely not suitable for spacecraft navigation due to their short lifetime (a few hundred hours [173]) as compared to typical exploration mission lifetimes.

Both the accuracy and lifetime issues of the conventional methods (emission lamps or absorption cells) may be addressed using laser frequency combs (LFC) [174], thus, creating the so-called “astro-comb” calibration approach. Great progress has been made in the last ten years in reducing the size and complexity of such systems [175,176]. While these systems remain complex and expensive, they are the topic of considerable contemporary research and are expected to be the frequency calibration method of choice for future systems requiring accuracy at the cm/s level. The technology will surely continue to improve in coming years. Regardless of the size and complexity of the LFC itself, the system still requires a stable frequency source—usually an oscillator referenced to a cesium or rubidium atomic clock. While atomic clocks suitable for space exploration may soon be available due to the DSAC [22], these are likely to remain expensive for the foreseeable future. Further, if one has an atomic clock, more straightforward autonomous navigation solutions may be available (e.g., one-way ranging with DSN [23]).

Thus, at the present, there appear to be no suitable sources for frequency calibration within the context of an autonomous navigation system. Specifically, emission lamps do not have an adequate lifetime, gas absorption cells do not have adequate accuracy, and astro-combs require an atomic clock.

#### 4.2.3. Measuring Spectral Shift with a Navigation Instrument

Significant technological advancement is required if spectral shift is to be used as a navigation observable. With the exception of technology demonstration missions, navigation instruments generally play a supporting role in space exploration. It is desirable for them to have a low mass, power, volume, and cost so that resources may be allocated to the mission’s scientific (or commercial) payload. Spectrographs capable of achieving radial velocity errors on cm/s level are not compatible with these needs.

Recent studies have presented complete error models for spectrographs used to produce stellar radial velocity measurements [177,178]. These complement empirical models, such as that of [179], which suggests the standard deviation of the radial velocity error is given by

(82) $$\sigma_{RV}\propto {\left(S/N\right)}^{-1}R^{-1}B^{-1}$$

where S/N is the SNR, *B* is the wavelength coverage, and R is the resolving power,

(83) $$R=\frac{\lambda }{\Delta \lambda }$$

A study of these models (both theoretical and empirical) clearly show the difficulties in obtaining radial velocity measurements having errors at the 10s of cm/s level with a spacecraft navigation instrument. Typical systems achieving better than 0.5 m/s have S/N>500, $R∼10^{5}$, and B∼300 nm, and a high-quality calibration source (see Section 4.2.2).

Achieving radial velocity errors below 1 m/s has only been achieved within the last 10–15 years, with these accomplishments making use of best-in-class facilities on the ground (not on a spacecraft) and requiring substantial investment. While new systems being proposed today (circa 2015–2019) are aiming for errors 1–30 cm/s [162], achieving such accuracy is not straightforward and is the topic of considerable worldwide effort. Consequently, even if the challenges of Section 4.2.1 and Section 4.2.2 are addressed, it seems doubtful that obtaining radial velocity measurements from stellar spectra with errors of <30 cm/s or better is possible within the typical size, mass, power, and cost requirements of a navigation instrument. In the author’s opinion, achieving the accuracies required for navigation within the Solar System using an acceptably small instrument will necessitate a fundamental change in the technique used to measure spectral shift, rather than an incremental improvement of existing spectrograph systems used in the search for exoplanets.

## 5. Instantaneous Estimation of Velocity from Simultaneous Star Sightings

The mathematical framework developed in Section 3 may now be deployed for the autonomous estimation of spacecraft velocity. Doing so requires some additional minor bookkeeping required by relativity. Once this is done, a framework is presented for instantaneous velocity estimation by either (1) measuring the absolute perturbation in the apparent direction of two stars or (2) measuring the perturbation in the apparent inter-star angles between four pairs of stars. The first method—perturbation in absolute star direction—is likely impossible to achieve in practice with modern technology for the reasons discussed at the end of Section 5.2. This method is still developed in detail, however, because it provides valuable insight that informs the second method. The second method—perturbation in inter-star angle—has numerous advantages and appears feasible with modern sensing technology (Section 4.1). Additionally, note that only StarNAV-SA measurements are considered from this point forward, as StarNAV-DE measurements are not desirable with contemporary astrophysical models and sensing technology (Section 4.2).

### 5.1. Representation of Spacecraft Velocity in Different Reference Frames

Within a general relativistic framework, representations of the position and velocity of a spacecraft vary depending on the reference system in which they are expressed by more than a simple Galilean transformation.

To begin, recognize that the BCRF velocity of a spacecraft, $\dot{r}$, may be related to the velocity measured by a fictitious stationary observer at the spacecraft’s location, v, according to [10]

(84) $$v=\left[1+\frac{\left(1+\gamma_{PPN}\right)\;U\left(r\right)}{c^{2}}\right]\dot{r}+O\left(c^{-4}\right)$$

where U(r) is the local gravitational potential of the solar system at the spacecraft’s location as given by Equation (50). The term $\gamma_{PPN}$ is from the parameterized post-Newtonian formalism and is very nearly unity [see discussion following Equation (33)].

While the relation from Equation (84) would be quite appropriate to navigate a vehicle in a heliocentric orbit (e.g., during an interplanetary transfer or a mission to certain small bodies), it is an inconvenient representation of the state to navigate a spacecraft in orbit about a planet. Navigation of a spacecraft in a bound orbit is generally performed in a planet-centered frame, rather than in a Solar System barycentric frame. Therefore, let the body-relative BCRF position of the spacecraft be given by $r_{sc}=r-r_{B}$. This body-relative state may be expressed in the BCRF ($r_{sc}$) or in a body-centered inertial frame whose axes are nominally aligned with BCRF ($\xi_{sc}$; e.g., a geocentric frame). Following the approach of [180,181], the BCRF body-relative velocity ${\dot{r}}_{sc}$ may be related to the corresponding velocity in the body-centered coordinate frame ${\dot{\xi }}_{sc}$ according to

(85) $${\dot{r}}_{sc}={\dot{\xi }}_{sc}-c^{-2}\left\{\left[\left(1+\gamma_{PPN}\right)U_{ex}\left(r_{B}\right)+\frac{1}{2}\left({\dot{r}}_{B}^{T}{\dot{r}}_{B}\right)+{\dot{r}}_{B}^{T}{\dot{\xi }}_{sc}\right]{\dot{\xi }}_{sc}+\frac{1}{2}\left({\dot{r}}_{B}^{T}{\dot{\xi }}_{sc}\right){\dot{r}}_{B}\right\}+O\left(c^{-4}\right)$$

where $U_{ex}\left(r_{B}\right)$ is the gravitational potential at $r_{B}$ excluding central body *B*. This agrees with the slightly more cumbersome result of [10] when the acceleration terms are neglected for the practical reasons described by [180].

Fortunately, the navigation accuracy of a purely StarNAV approach is not sufficiently good to require a relativistic treatment within the velocity estimation framework—ultimately allowing the replacement of Equations (84) and (85) with their substantially simpler Newtonian (classical) counterparts. This fact is not self-evident given the central role relativity plays elsewhere in this work and is now justified.

The key conclusion from the discussions that follow Equations (43) and (47) is that only terms of $O(∥\beta {∥}^{2})$ need be considered when determining the change in apparent star direction in situations where the sensor error is larger than a few μas, as is certainly the case for practical real-time navigation for the foreseeable future. That is, terms of $O(∥\beta {∥}^{3})$ and smaller are neglected. Recalling that β=v/c from Equation (38), terms of $O(∥\beta {∥}^{3})$ are equivalent to terms of $O(c^{-3})$. Consequently, for the task at hand, it is sufficient to ignore velocity perturbation terms of $O(c^{-2})$

(86) $$v=\dot{r}+O\left(c^{-2}\right)={\dot{r}}_{B}+{\dot{\xi }}_{sc}+O\left(c^{-2}\right)$$

since $O(c^{-2})$ terms in velocity become $O(c^{-3})$ terms in β

(87) $$\beta =v/c=\dot{r}/c+O\left(c^{-3}\right)=c^{-1}\left({\dot{r}}_{B}+{\dot{\xi }}_{sc}\right)+O\left(c^{-3}\right)$$

Therefore, for the purposes of velocity estimation by stellar aberration, it usually is acceptable to neglect the relativistic effects when representing velocity in different frames and simply use the relations one would expect from Newtonian physics

(88) $$\dot{r}\approx v$$

(89) $${\dot{\xi }}_{sc}\approx {\dot{r}}_{sc}\approx v-{\dot{r}}_{B}$$

### 5.2. Instantaneous Velocity Fix from Perturbation in Absolute Star Directions

This section develops a solution method for an instantaneous velocity fix assuming one can obtain absolute star direction measurements $u_{i}^{\prime \prime }$ on the order of 0.1–1 mas. As was shown in Section 3.2.2.1, measurement accuracy at this level requires a second-order expansion of β. Therefore, keeping only the necessary terms from Equation (43),

(90) $$\begin{array}{c} u_{i}^{\prime \prime }=u_{i}^{\prime }+u_{i}^{\prime }\times \left(\beta \times u_{i}^{\prime }\right)-\left[\left({u_{i}^{\prime }}^{T}\beta \right)u_{i}^{\prime }\times \left(\beta \times u_{i}^{\prime }\right)+\left(1/2\right)\beta \times \left(u_{i}^{\prime }\times \beta \right)\right]+{O(∥\beta ∥}^{3}) \end{array}$$

which after substitution of Equations (29) and (38) may be rewritten as

(91) $$\begin{array}{c} u_{i}^{\prime \prime }=u_{i}+\delta u_{i}-\frac{1}{c}{\left[u_{i}\times \right]}^{2}v+\frac{1}{c^{2}}\left[\left(u_{i}^{T}v\right)\left[u_{i}\times \right]-\frac{1}{2}\left[v\times \right]\right]\left[u_{i}\times \right]v+O\left(c^{-3}\right) \end{array}$$

where, repeating Equation (33) and letting $\gamma_{PPN}=1$, the gravitational deflection term $\delta u_{i}$ is $O(c^{-2})$ and is given by

(92) $$\delta u_{i}=-\sum_{B}\frac{2GM_{B}}{c^{2}{∥d_{iB}∥}^{2}}\left(1+u_{i}^{T}u_{B}\right)d_{iB}$$

For the sake of discussion, consider a spacecraft in Earth orbit. In this case, taking advantage of the results form Table 2, it is possible to rewrite $d_{iB}$ from Equation (34) for all bodies other than the Earth and Moon in terms of *a priori* known quantities from the star catalog [77] and ephemeris files [182],

(93) $$d_{iB}\approx {\tilde{d}}_{iB}=-{\left[u_{i}\times \right]}^{2}\left(r_{B}-r_{E}\right)$$

Since the Moon has a relatively small exclusion angle (Table 2), it can be ignored altogether by the judicious selection of guide stars. Thus, the gravitational deflection term $\delta u_{i}$ may be split into unknown terms (Earth) and known terms (Sun, Jupiter, Saturn)

(94) $$\begin{array}{c} \delta u_{i}\approx -\frac{2GM_{E}}{c^{2}{∥d_{iE}∥}^{2}}\left(1+u_{i}^{T}u_{E}\right)d_{iE}-\sum_{B\neq E,M}\frac{2GM_{B}}{c^{2}{∥{\tilde{d}}_{iB}∥}^{2}}\left(1+u_{i}^{T}u_{B}\right){\tilde{d}}_{iB} \end{array}$$

A similar approach may be taken for a spacecraft orbiting a different planet by selecting the appropriate division of known and unknown terms for that particular situation.

Further, substituting from Equation (35), the term regarding Earth may be rewritten yet again as

(95) $$\begin{array}{c} \frac{2GM_{E}}{c^{2}{∥d_{iE}∥}^{2}}\left(1+u_{i}^{T}u_{E}\right)d_{iE}=\frac{1}{c}\alpha \cot\left(\theta_{iE}/2\right)w_{iE} \end{array}$$

where the new unknown scalar α is the same for all concurrent star observations

(96) $$\begin{array}{c} \alpha =\frac{2GM_{E}}{c\rho_{E}} \end{array}$$

It is presumed that $\theta_{iE}$ and $w_{iE}$ are known in all practical scenarios since they only depend on the direction from the spacecraft to Earth (see Equations (32) and (36)), which can easily be determined to suitable accuracy with existing OPNAV techniques [3] or a horizon sensor [183].

Therefore, grouping known terms on the left and unknown terms on the right,

(97) $$\begin{array}{c} u_{i}^{\prime \prime }-u_{i}-\delta u_{ex_{i}}=-\frac{1}{c}\left[{\left[u_{i}\times \right]}^{2}v+\alpha \cot\left(\theta_{iE}/2\right)w_{iE}\right]+\frac{1}{c^{2}}\left[\left({u_{i}}^{T}v\right)\left[u_{i}\times \right]-\frac{1}{2}\left[v\times \right]\right]\left[u_{i}\times \right]v \end{array}$$

where

(98) $$\begin{array}{c} \delta u_{ex_{i}}=-\sum_{B\neq E,M}\frac{2GM_{B}}{c^{2}{∥{\tilde{d}}_{iB}∥}^{2}}\left(1+u_{i}^{T}u_{B}\right){\tilde{d}}_{iB} \end{array}$$

The only unknowns in Equation (97) are the 3×1 velocity v and the scalar α. Clearly, an instantaneous solution cannot be obtained from only one star LOS measurement, since velocity in the direction of $u_{i}$ creates no stellar aberration. A unique solution for v and α exists for simultaneous measurements to any set of two (or more) stars whose LOS directions are not (nearly) collinear. The only remaining step, therefore, is to solve the system of 3n equations (for n≥2) that are linear in the unknown α and quadratic in the unknown v. This can be achieved to $O(∥\beta {∥}^{3})$—corresponding to neglected stellar aberration effects on the order of 0.1 μas—through a single step of successive substitution.

Therefore, proceed by solving the following linear system in the least squares sense with an initial guess of ${\dot{r}}_{sc_{\left(0\right)}}=0_{3\times 1}$ such that $v_{\left(0\right)}={\dot{r}}_{E}$

(99) $$\left[\begin{array}{c} u_{1}^{\prime \prime }-u_{1}-\delta u_{ex_{1}}\\ u_{2}^{\prime \prime }-u_{2}-\delta u_{ex_{2}}\\ \vdots \\ u_{n}^{\prime \prime }-u_{n}-\delta u_{ex_{n}} \end{array}\right]=\left[\begin{array}{cc} H_{v_{1}} & H_{\alpha_{1}}\\ H_{v_{2}} & H_{\alpha_{2}}\\ \vdots & \vdots \\ H_{v_{n}} & H_{\alpha_{n}} \end{array}\right]\left[\begin{array}{c} v_{\left(k\right)}\\ \alpha \end{array}\right]$$

where

(100) $$H_{v_{i}}=\frac{1}{c}\left\{\frac{1}{c}\left(\left(u_{i}^{T}v_{\left(k-1\right)}\right)\left[u_{i}\times \right]-\frac{1}{2}\left[v_{\left(k-1\right)}\times \right]\right)-\left[u_{i}\times \right]\right\}\left[u_{i}\times \right]$$

(101) $$H_{\alpha_{i}}=-\frac{1}{c}\cot\left(\theta_{iE}/2\right)w_{iE}$$

The above equation is evaluated twice until one obtains the estimate for BCRF the velocity as $\hat{\dot{r}}=\hat{v}=v_{\left(2\right)}$. Thus, following Equation (89), the estimated velocity of the spacecraft relative to Earth is ${\dot{r}}_{sc}=v_{\left(2\right)}-{\dot{r}}_{E}$.

**Figure 10:**
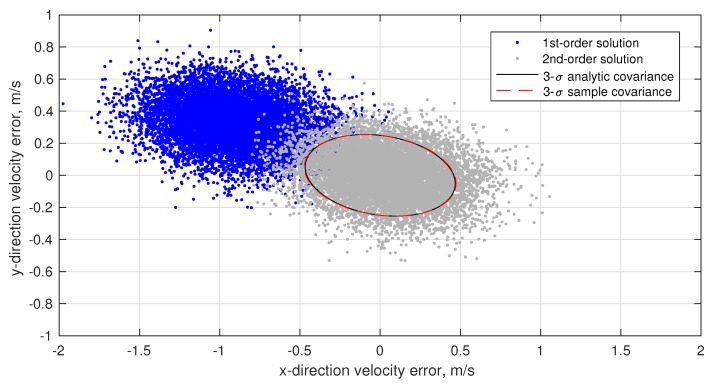
Instantaneous velocity estimate residuals (10,000 Monte Carlo cases) for spacecraft in geostationary orbit using observation to two stars with a bearing error to each of $\sigma_{\phi_{i}}=0.1$ mas.

Observe that an evaluation of Equation (99) when $v_{\left(0\right)}=0_{3\times 1}$ is equivalent to solving the problem using only first-order terms in v. It is instructive to consider the implications of this, as it highlights the need for the inclusion of second-order terms. A comparison of the first-order and second-order solutions for an example spacecraft in geostationary orbit is shown in Figure 10 (for the *x*–*y* plane only). The bias in the first-order solution is due to the neglected terms of $O(c^{-2})$, and is almost entirely removed by inclusion of these $O(c^{-2})$ terms in the second-order solution. This example assumes simultaneous observations of two stars separated by $\theta_{ij}=90$ deg, with each star observation having a bearing error of $\sigma_{\phi_{i}}=0.1$ mas. The truth model uses the complete non-linear stellar aberration expression (Equation (42)) and includes the gravitational deflection of light from the Earth, Sun, and Jupiter. Estimation of v and α is performed following the procedure and approximations described in the preceding discussion.

Figure 10 also shows the numerically-computed 3σ sample covariance, which agrees well with the analytic covariance. The analytic covariance is computed as usual for a least square solution. The statistics of individual star sightings are assumed to follow the QUEST measurement model [184,185]

(102) $$R_{u_{i}^{\prime \prime }}=E\left[\left(u_{i}^{\prime \prime }-E\left[u_{i}^{\prime \prime }\right]\right){\left(u_{i}^{\prime \prime }-E\left[u_{i}^{\prime \prime }\right]\right)}^{T}\right]\approx \sigma_{\phi_{i}}^{2}\left(I_{3\times 3}-u_{i}^{\prime \prime }{u^{\prime \prime }}_{i}^{T}\right)$$

where $\sigma_{\phi_{i}}$ is the standard deviation of the angular error for a particular star sighting. The constraint $∥u_{i}^{\prime \prime }∥=1$ leads the covariance matrix $R_{u_{i}^{\prime \prime }}$ to be singular, which is problematic since the computation of covariance generally requires $R_{u_{i}^{\prime \prime }}^{-1}$. Following an approach similar to [185] it is possible to show that

(103) $$P\approx {\left(\sum_{i=1}^{n}\frac{1}{\sigma_{\phi_{i}}^{2}}\left[\begin{array}{cc} H_{v_{i}}^{T}H_{v_{i}} & H_{v_{i}}^{T}H_{\alpha_{i}}\\ H_{\alpha_{i}}^{T}H_{v_{i}} & H_{\alpha_{i}}^{T}H_{\alpha_{i}} \end{array}\right]\right)}^{-1}$$

**Figure 11:**
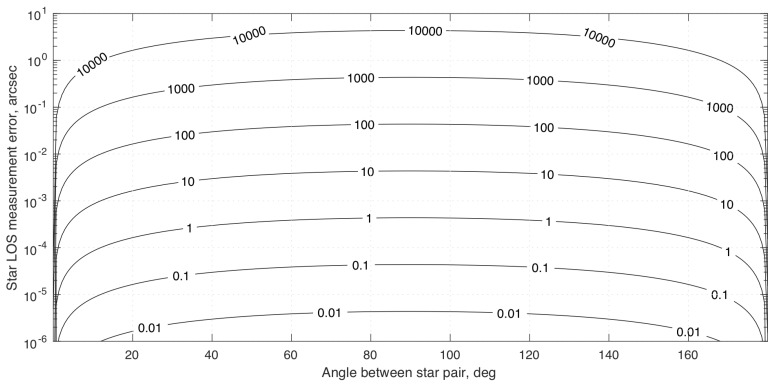
Contours of total instantaneous velocity error (m/s) using absolute stellar aberration of a single star pair.

When measuring the absolute stellar aberration, a solution for v is possible with two or more star measurements. Therefore consider two stars with an inter-star angle of $\theta_{ij}$ that varies from 0 to 180 deg. Additionally, let the standard deviation for each LOS measurement vary from $\sigma_{\phi_{i}}=1$μas to $\sigma_{\phi_{i}}=10$ arcsec. For each combination of $\theta_{ij}$ and σ, the total velocity error is computed as $\sqrt{\mathrm{tr}\left[P_{vv}\right]}$ and the results are shown in Figure 11. The best performance is achieved when $\theta_{ij}=90$ deg and $\sigma_{\phi_{i}}$ is as small as possible.

Real-time measurement of apparent star direction as expressed in the BCRF below at the 1 mas level (or below) is difficult. While 1 mas accuracy may be possible in the sensor or spacecraft frame, the absolute direction of $u_{i}^{\prime \prime }$ at is likely not knowable in real-time to better than about 1 arcsec (or worse in the complete absence of *a priori* state knowledge).

### 5.3. Instantaneous Velocity Fix from Perturbation of Inter-Star Angle

The use of inter-star angles removes the need for precise knowledge of the sensor’s absolute orientation. Thus, although absolute LOS measurements better than 1 mas are difficult, such real-time precision may be possible when measuring inter-star angles (see Section 4.1).

Obtaining an instantaneous velocity fix using inter-star angle measurements with errors below 1 mas requires a second order expansion in β. Therefore, retaining the appropriate terms from Equation (47),

(104) $$\begin{array}{c} {u_{i}^{\prime \prime }}^{T}u_{j}^{\prime \prime }={u_{i}^{\prime }}^{T}u_{j}^{\prime }+\left(1-{u_{i}^{\prime }}^{T}u_{j}^{\prime }\right)\left\{\left[\beta^{T}u_{i}^{\prime }+\beta^{T}u_{j}^{\prime }\right]-\left[{\left(\beta^{T}u_{i}^{\prime }\right)}^{2}+{\left(\beta^{T}u_{j}^{\prime }\right)}^{2}+\left(\beta^{T}u_{i}^{\prime }\right)\left(\beta^{T}u_{j}^{\prime }\right)-\beta^{T}\beta \right]\right\}+{O(∥\beta ∥}^{3}) \end{array}$$

which may be rewritten as

(105) $$\begin{array}{c} {u_{i}^{\prime \prime }}^{T}u_{j}^{\prime \prime }=u_{i}^{T}u_{j}+u_{i}^{T}\delta u_{j}+u_{j}^{T}\delta u_{i}+\frac{1}{c}\left(1-u_{i}^{T}u_{j}\right)\left[\left(u_{i}^{T}+u_{j}^{T}\right)v-\frac{1}{c}v^{T}A_{ij}v\right]+O\left(c^{-3}\right) \end{array}$$

where the quadratic form of $v^{T}A_{ij}v$ allows $A_{ij}$ to be written as a symmetric matrix

(106) $$A_{ij}=u_{i}u_{i}^{T}+u_{j}u_{j}^{T}+\frac{1}{2}\left(u_{i}u_{j}^{T}+u_{j}u_{i}^{T}\right)-I_{3\times 3}$$

As before, consider a spacecraft in Earth orbit such that $\delta u_{i}$ may be split according to Equation (94). Now, grouping known terms on the left-hand side and unknown terms on the right-hand side, Equation (105) becomes

(107) $$\begin{array}{cc} {u_{i}^{\prime \prime }}^{T}u_{j}^{\prime \prime }-u_{i}^{T}u_{j}-u_{i}^{T}\delta u_{ex_{j}}-u_{j}^{T}\delta u_{ex_{i}}= & -\alpha \frac{1}{c}\left[\cot\left(\theta_{iE}/2\right)u_{j}^{T}w_{iE}+\cot\left(\theta_{jE}/2\right)u_{i}^{T}w_{jE}\right]\\ +\frac{1}{c}\left(1-u_{i}^{T}u_{j}\right)\left(u_{i}^{T}+u_{j}^{T}-\frac{1}{c}v^{T}A_{ij}\right)v \end{array}$$

Therefore, proceed by solving the following linear system in the least squares sense with an initial guess of ${\dot{r}}_{sc_{\left(0\right)}}=0_{3\times 1}$ such that $v_{\left(0\right)}={\dot{r}}_{E}$

(108) $$\left[\begin{array}{c} {u_{i}^{\prime \prime }}^{T}u_{j}^{\prime \prime }-u_{i}^{T}u_{j}-u_{i}^{T}\delta u_{ex_{j}}-u_{j}^{T}\delta u_{ex_{i}}\\ \vdots \\ {u_{p}^{\prime \prime }}^{T}u_{\ell }^{\prime \prime }-u_{p}^{T}u_{\ell }-u_{p}^{T}\delta u_{ex_{\ell }}-u_{\ell }^{T}\delta u_{ex_{p}} \end{array}\right]=\left[\begin{array}{cc} H_{v_{ij}} & H_{\alpha_{ij}}\\ \vdots & \vdots \\ H_{v_{p\ell }} & H_{\alpha_{p\ell }} \end{array}\right]\left[\begin{array}{c} v_{\left(k\right)}\\ \alpha \end{array}\right]$$

where

(109) $$H_{v_{ij}}=\frac{1}{c}\left(1-u_{i}^{T}u_{j}\right)\left(u_{i}^{T}+u_{j}^{T}-\frac{1}{c}v_{\left(k-1\right)}^{T}A_{ij}\right)$$

(110) $$H_{\alpha_{ij}}=-\frac{1}{c}\left[\cot\left(\theta_{iE}/2\right)u_{j}^{T}w_{iE}+\cot\left(\theta_{jE}/2\right)u_{i}^{T}w_{jE}\right]$$

The above equations are evaluated twice to obtain the BCRF velocity $\hat{\dot{r}}=\hat{v}=v_{\left(2\right)}$. Thus, following Equation (89), the estimated velocity of the spacecraft relative to Earth is ${\dot{r}}_{sc}=v_{\left(2\right)}-{\dot{r}}_{E}$.

**Figure 12:**
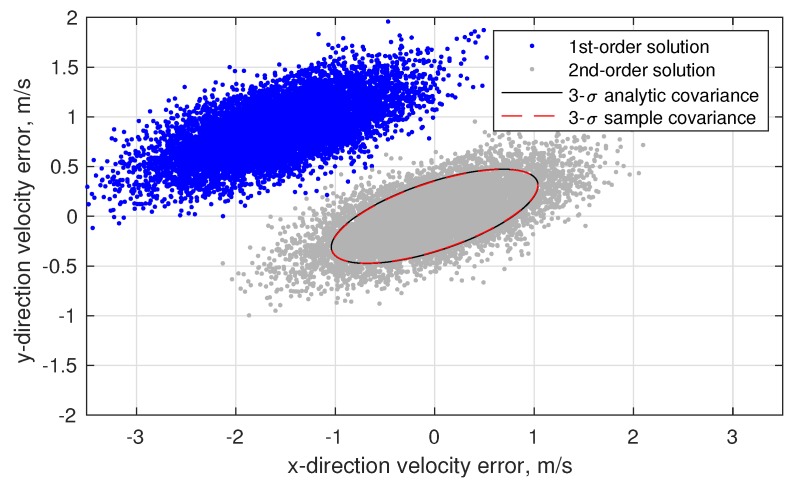
Instantaneous velocity estimate residuals (10,000 Monte Carlo cases) for spacecraft in geostationary orbit using the inter-star angles between four stars. Bearing error to each star is $\sigma_{\phi_{i}}\;=\;0.1$ mas.

To see the importance of including the second-order terms in v for instantaneous velocity estimation using inter-star angles, consider a spacecraft in GEO that measures the direction to four stars. Individual star direction measurements in the sensor frame are assumed to follow the QUEST measurement model and then inter-star angles are computed from these measurements. The orientation-independent geometry of these four star directions is entirely described by five inter-star angles. Although 42=6, only five of the angles are independent. Including all six inter-star angles adds no new information and results in a rank deficient measurement covariance matrix. In this example, the first three star sightings are approximately orthogonal to one another and the fourth star has an inter-star angle of about 55 deg with respect to each of the first three. Assuming a bearing error of $\sigma_{\phi_{i}}=0.1$ mas, representative instantaneous velocity estimation performance is as shown in Figure 12. These results include the gravitational deflection of starlight from the Sun, Earth, and Jupiter.

The analytic covariance from Figure 12 is computed as follows. To begin, consider the star pair that includes star *i* and star *j* and define $y_{ij}$ to be

(111) $$y_{ij}=\cos\theta_{ij}^{\prime \prime }={u_{i}^{\prime \prime }}^{T}u_{j}^{\prime \prime }$$

such that

(112) $$\delta y_{ij}={u_{i}^{\prime \prime }}^{T}\delta u_{j}^{\prime \prime }+{u_{j}^{\prime \prime }}^{T}\delta u_{i}^{\prime \prime }$$

Consequently, the diagonal terms in the covariance matrix are given by

(113) $$\sigma_{ij,ij}^{2}=E\left[\delta y_{ij}^{2}\right]={u_{i}^{\prime \prime }}^{T}R_{u_{j}^{\prime \prime }}u_{i}^{\prime \prime }+{u_{j}^{\prime \prime }}^{T}R_{u_{i}^{\prime \prime }}u_{j}^{\prime \prime }$$

where $R_{u_{i}}$ is the QUEST measurement model from Equation (102) and the errors in the individual star LOS measurements are assumed to be uncorrelated with one another (i.e., $E[\delta u_{i}^{\prime \prime }\delta {u_{j}^{\prime \prime }}^{T}]=0$). Likewise, the off-diagonal terms are given by

(114) $$\sigma_{ij,i\ell }^{2}=\sigma_{i\ell ,ij}^{2}=E\left[\delta y_{ij}\delta y_{i\ell }\right]={u_{j}^{\prime \prime }}^{T}R_{u_{i}^{\prime \prime }}u_{\ell }^{\prime \prime }$$

These may be combined to find the fully correlated covariance matrix R for an arbitrary combination of inter-star angles constructed from a given set of LOS measurements. From here, the analytic covariance (as shown by the black line in Figure 12) may be computed as

(115) $$P={\left(H^{T}R^{-1}H\right)}^{-1}$$

**Figure 13:**
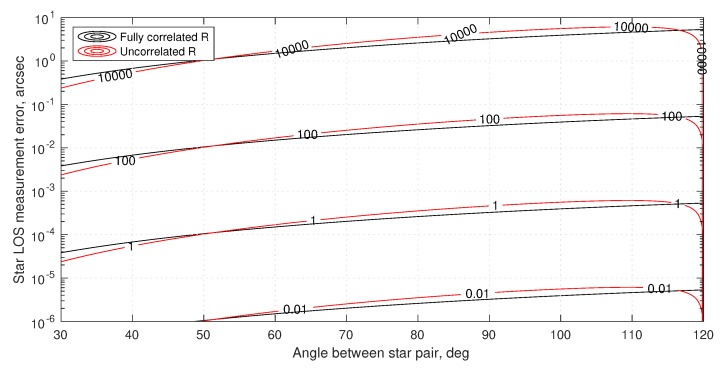
Contours of total instantaneous velocity error (m/s) using inter-star stellar aberration from three stars.

This analytic covariance may also be used to better understand the sensitivity of the StarNAV velocity fix to inter-star angle. Momentarily ignoring the gravitational deflection of light (which introduces a small dependence on location), consider three stars separated from one another by the angle θ. In this case, the covariance of the velocity estimate is simply

(116) $$P_{vv}\approx {\left(H_{v}^{T}R^{-1}H_{v}\right)}^{-1}$$

where $H_{v}$ and R are as discussed above. Observe that rank($H_{v}$) = 3 for 0 deg <θ< 120 deg, although numerical conditioning of the problem degrades as one approaches the endpoints (with rank($H_{v}$) = 0 at exactly θ=0 deg and rank($H_{v}$) = 2 at exactly θ=120 deg). Therefore, considering all possible three-star pyramids with inter-star angles from 0–120 deg, one may compute the total velocity error as $\sqrt{\mathrm{tr}[P_{vv}]}$. Contours of this velocity error are shown in Figure 13 using the fully correlated R from above (in black) as well as the uncorrelated measurement covariance $R=\sigma_{\theta }^{2}I_{3\times 3}$ (in red). In reality, an uncorrelated R would only occur if six stars were observed and each of the three star pairs used unique stars (thus, eliminating correlation between two measured inter-star angles, e.g., $\theta_{ij}^{\prime \prime }$ and $\theta_{p\ell }^{\prime \prime }$).The two covariance expressions produce velocity estimate errors of a similar magnitude, with larger inter-star angles generally being better.

Of note is that the uncorrelated R reaches a maximum at $\cos(\theta_{ij})=-1/3$, which is equivalent to $\theta_{ij}=109.47$ deg. This is the inter-star angle where each row of $H_{v}$ has the maximum length (and, therefore, produces the most sensitivity in inter-star angle to a perturbation in velocity). That an inter-star angle of 109.47 deg produces maximum sensitivity to velocity perturbations was also observed by [9].

Furthermore, the novel observation is now made that θ=109.47 deg is the inter-star angle leading to the star direction bisectors being orthogonal to one another in the case of the three-star pyramid. Note that each row of $H_{v}$ is in the direction $u_{i}+u_{j}$, which is in the direction of the bisector of the two contributing star directions. Thus, by choosing a star pyramid with $\theta_{ij}=109.47$, one is also choosing the special angle that makes the rows of $H_{v}$ orthogonal to one another.

## 6. Initial Orbit Determination (IOD) using StarNAV

Three or more velocity vector measurements—such as those found using the instantaneous StarNav velocity fix from Section 5.3—uniquely define an orbit for a spacecraft obeying Keplerian dynamics. The concept of an analytic initial orbit determination (IOD) solution from three velocity vectors was first posited in [1] and an elegant geometric solution was developed shortly after in [2].

Solving the IOD problem with three velocity vector measurements is analogous to the classical Gibbs Problem [186,187,188] where the knowns and unknowns have been switched. In the Gibbs problem, which was first solved by J. W. Gibbs using vector analysis in 1889 [186], one is given three position vectors (each with an unknown velocity) and must solve for the velocity corresponding to one of those positions to fully define the orbit. In the velocity-only IOD problem, one is given three velocity vectors (each with an unknown position) and must solve for the position corresponding to one of those velocities to fully define the orbit. Note that the velocity-only IOD problem is not an alternative to Gibbs problem (the knowns and unknowns are different, so the problems to which they are applicable are rarely interchangeable), it’s just that the two problems have similar structure.

### 6.1. Analytic Velocity-Only IOD Solution using Geometry of the Orbital Hodograph

**Figure 14:**
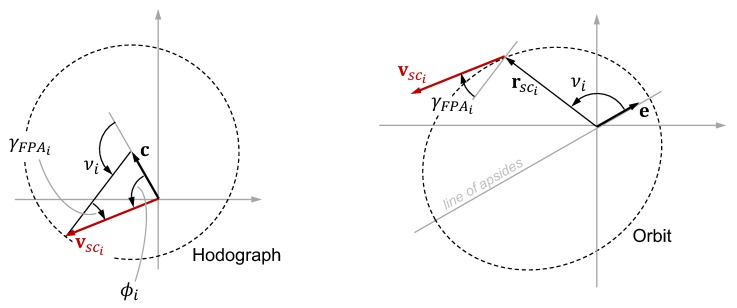
Geometric relationship between the orbital hodograph (**left**) and an elliptical orbit (**right**).

The StarNAV IOD solution technique presented here follows the velocity-only IOD strategy outlined in [2], which makes use of the orbital hodograph. Developed by Hamilton in 1847 [189], the hodograph is the locus of points traced by a trajectory’s moving velocity vector while keeping the tail fixed. As Hamilton observed in his original work, this curve is a perfect circle for any body undergoing Keplerian motion—regardless of the conic section describing the path of the actual orbit (i.e., circular, elliptical, parabolic, or hyperbolic). An example is shown in Figure 14. Despite its relative obscurity, the beautiful geometry of the hodograph has made it a powerful tool for solving many practical engineering problems in spaceflight dynamics [2,190,191,192,193].

In the absence of noise or perturbations from two-body motion, all of the velocity vectors will lie entirely within the orbital plane. Therefore, assuming three or more StarNAV velocity measurements obtained as in Section 5.3, proceed by finding the unit normal to the orbital plane as the solution to the following linear system [1]

(117) $$\left[\begin{array}{c} v_{sc_{1}}^{T}\\ v_{sc_{2}}^{T}\\ \vdots \\ v_{sc_{n}}^{T} \end{array}\right]k=0_{n\times 1}$$

where ∥k∥=1. The solution for k may be found in the total least squares (TLS) sense [194] through a singular value decomposition (SVD). Care must be taken to ensure that k is in the direction of the orbit’s specific angular momentum, as discussed at length in [1,2].

The orbit plane unit normal k may be used to construct a new orbit frame. Define $u_{x}$ as

(118) $$u_{x}=\langle v_{sc_{1}}\times k\rangle$$

where, once again, $\langle \cdot \rangle$ denotes vector length normalization. Let $u_{y}$ be chosen to complete the right-handed system $\left\{u_{x},u_{y},k\right\}$ describing the coordinate axes of the orbit frame. Therefore, letting $T_{O}^{I}$ be the matrix that rotates a vector from the inertial frame to the orbit frame, one finds that

(119) $$T_{O}^{I}=\left[\begin{array}{c} u_{x}^{T}\\ u_{y}^{T}\\ k^{T} \end{array}\right]$$

Consequently, orthographic projection of the measured velocity vectors onto the orbital plane may may be performed as

(120) $$\left[\begin{array}{c} {\dot{x}}_{O_{i}}\\ {\dot{y}}_{O_{i}} \end{array}\right]=\left[\begin{array}{ccc} 1 & 0 & 0\\ 0 & 1 & 0 \end{array}\right]T_{O}^{I}v_{sc_{i}}$$

Since the hodograph forms a perfect circle, the next step is to fit a circle to the projected velocity vectors in the orbit plane. This may be done analytically through any number of commonly available circle fitting algorithms [195]. This work chooses to make use of an algebraic circle fitting approach to permit a non-iterative solution. If the hodograph circle is defined by its center coordinates $\left\{{\dot{x}}_{c},{\dot{y}}_{c}\right\}$ and its radius *R*, then the best fit may be found in the least squares sense as the solution to the following linear system [2]

(121) $$\left[\begin{array}{ccc} 2{\dot{x}}_{O_{1}} & 2{\dot{y}}_{O_{1}} & -1\\ 2{\dot{x}}_{O_{2}} & 2{\dot{y}}_{O_{2}} & -1\\ \vdots & \vdots & \vdots \\ 2{\dot{x}}_{O_{n}} & 2{\dot{y}}_{O_{n}} & -1 \end{array}\right]\left[\begin{array}{c} {\dot{x}}_{c}\\ {\dot{y}}_{c}\\ g \end{array}\right]=\left[\begin{array}{c} {\dot{x}}_{O_{1}}^{2}+{\dot{y}}_{O_{1}}^{2}\\ {\dot{x}}_{O_{2}}^{2}+{\dot{y}}_{O_{2}}^{2}\\ \vdots \\ {\dot{x}}_{O_{n}}^{2}+{\dot{y}}_{O_{n}}^{2} \end{array}\right]$$

where the hodograph circle radius is found making use of the intermediate variable *g*

(122) $$R=\sqrt{{\dot{x}}_{c}^{2}+{\dot{y}}_{c}^{2}-g}$$

The location of the hodograph circle center may now be transformed from the orbit frame back to the inertial frame according to

(123) $$c={\left(T_{O}^{I}\right)}^{T}\left[\begin{array}{c} {\dot{x}}_{c}\\ {\dot{y}}_{c}\\ 0 \end{array}\right]$$

In some cases it may be useful to apply these results to find the orbit’s eccentricity vector, which may be computed as

(124) $$e=\frac{c}{R}\times k$$

With the hodograph fully defined, one only needs to apply simple geometry to obtain the position vector corresponding to any one of the velocity vectors. This may be done in three steps.

First, compute the unit vector describing the direction from the center of the planet to the spacecraft,

(125) $$\langle r_{sc_{i}}\rangle =\langle v_{sc_{i}}-c\rangle \times k$$

Renormalization of the right-hand-side to ensure the result is a unit vector is only necessary with noisy measurements, since $\langle v_{sc_{i}}-c\rangle$ is perpendicular to k by construction in the noise-free situation.

Second, compute the component of velocity that lies in the plane perpendicular to the direction from the planet to the spacecraft

(126) $$v_{sc{⊥}_{i}}=\left(I_{3\times 3}-\langle r_{sc_{i}}\rangle {\langle r_{sc_{i}}\rangle }^{T}\right)v_{sc_{i}}=-{\left[\langle r_{sc_{i}}\rangle \times \right]}^{2}v_{sc_{i}}$$

Third, and finally, compute the magnitude of $r_{sc_{i}}$. It was suggested in [2] that $\rho_{sc_{i}}=∥r_{sc_{i}}∥$ be computed as

(127) $$\rho_{sc_{i}}=∥r_{sc_{i}}∥=\frac{\mu ∥e+\langle r_{sc_{i}}\rangle ∥}{∥v_{sc{⊥}_{i}}∥\;∥v_{sc_{i}}∥}$$

where e is the orbit’s eccentricity vector and μ is the central body’s gravitational parameter. Recognizing, however, that the magnitude of the specific angular momentum, h=∥h∥, may be written in terms of the hodograph circle radius, *R*,

(128) $$h=\frac{\mu }{R}$$

it is observed here that $\rho_{sc_{i}}$ is more straightforwardly computed (and with no need for computation of e) as

(129) $$\rho_{sc_{i}}=\frac{\mu }{R∥v_{sc{⊥}_{i}}∥}$$

Therefore, the position vector corresponding to the velocity $v_{sc_{i}}$ is given by combining the results of Equations (125) and (129)

(130) $$r_{sc_{i}}=\rho_{sc_{i}}\langle r_{sc_{i}}\rangle$$

The detailed derivation for all of the above (with the exception of the new approach for finding $\rho_{sc_{i}}$) may be found in [2].

### 6.2. Improved IOD using Many Velocity Vectors Collected at Known Times

While algebraically exact in the absence of measurement noise, the trouble with the final portion of the solution from [2] (as summarized in Equations (125)–(130)) is that the position estimate inherits all of the error associated with its corresponding velocity vector. Substantially better performance may be obtained by estimating the orbit as a whole and then finding the spacecraft position at the appropriate time. This allows the position estimate at time $t_{k}$ to more fully benefit from the information contained in the velocity measurements obtained at other times. With the exception of solving Kepler’s equation, the improved solution procedure remains otherwise non-iterative.

Observe from hodograph geometry (Figure 14) that the true anomaly $\nu_{i}$ may be written explicitly in terms of $v_{sc_{i}}$ and the hodograph fit parameters

(131) $$∥v_{sc_{i}}∥\cos\left(\phi_{i}\right)=∥c∥+R\cos\left(\nu_{i}\right)$$

where cos(ϕ) may be found from the law of cosines

(132) $$R^{2}=∥v_{sc_{i}}{∥}^{2}+{∥c∥}^{2}-2∥v_{sc_{i}}∥\;∥c∥\cos\left(\phi_{i}\right)$$

While it may be tempting to combine these equations to solve for the unknown $\nu_{i}$, doing so is generally inadvisable. Although exact for perfect observations, the presence of noise in practical StarNAV measurements of $v_{sc_{i}}$ creates problems with the explicit use of Equations (131) and (132) near periapsis, where it is not uncommon to find $∥v_{sc_{i}}∥>(∥c∥+R)$ for some measurements. Much better numerical results are achievable by only using the direction of the velocity vector, $\langle v_{sc_{i}}\rangle$.

Therefore, proceed by recognizing that

(133) $$∥\sin\left(\phi_{i}\right)∥=∥\langle c\rangle \times \langle v_{sc_{i}}\rangle ∥$$

(134) $$\cos\left(\phi_{i}\right)={\langle c\rangle }^{T}\langle v_{sc_{i}}\rangle$$

and, from the law of sines,

(135) $$\frac{∥\sin(\phi_{i})∥}{R}=\frac{∥\sin(\nu_{i}-\phi_{i})∥}{∥c∥}$$

The correct quadrant for $\nu_{i}$ may be determined from the direction of $\langle c\rangle \times \langle v_{sc_{i}}\rangle$ relative to the direction of the orbit’s specific angular momentum vector. Under the assumption that $\phi_{i}$ is computed as

(136) $$\phi_{i}=\arccos\left({\langle c\rangle }^{T}\langle v_{sc_{i}}\rangle \right)\in \left[0,\pi \right]$$

the correct value for the true anomaly is

(137) $$\nu_{i}=\left\{\begin{array}{cc} \phi_{i}+\arcsin\left(\frac{1}{R}∥c\times \langle v_{sc_{i}}\rangle ∥\right) & \mathrm{for}\;{\left(\langle c\rangle \times \langle v_{sc_{i}}\rangle \right)}^{T}k>0\\ 2\pi -\phi_{i}-\arcsin\left(\frac{1}{R}∥c\times \langle v_{sc_{i}}\rangle ∥\right) & \mathrm{otherwise} \end{array}\right.$$

where $\nu_{i}\in \left[0,2\pi \right]$.

With the true anomaly known at each time, the objective is now to find the time of periapsis passing suggested by each individual velocity measurement. Begin by using the true anomaly computed by Equation (137) to find the eccentric anomaly at those same times ($E_{i}$)

(138) $$\sin\left(E_{i}\right)=\frac{\sin\left(\nu_{i}\right)\sqrt{1-e^{2}}}{1+e\cos(\nu_{i})}$$

(139) $$\cos\left(E_{i}\right)=\frac{e+\cos(\nu_{i})}{1+e\cos(\nu_{i})}$$

where e=∥e∥ and is found using Equation (124). This, in turn, may be used to find the corresponding mean anomaly ($M_{i}$) from Kepler’s equation [187]

(140) $$M_{i}=E_{i}-e\sin\left(E_{i}\right)$$

Further recalling that $M_{i}$ is related to time from the last periapsis passing by

(141) $$M_{i}=\sqrt{\frac{\mu }{a^{3}}}\left(t_{i}-t_{p_{i}}\right)$$

it is straightforward to find mean time of the periapsis passing before the first measurement as

(142) $$t_{p_{0}}=\frac{1}{n}\sum_{i=1}^{n}\left\{t_{i}-\sqrt{\frac{a^{3}}{\mu }}\left[2\pi k_{i}+E_{i}-e\sin\left(E_{i}\right)\right]\right\}$$

where $k_{i}$ is the number of integer passings through the periapsis that occur between the first measurement time and time $t_{i}$. This is simple to keep track of with the available data.

With the orbit fully defined and a reference time for a periapsis passing, it is now possible to compute the spacecraft location at any given time. This is straightforward Keplerian orbit analysis covered in any introductory text on astrodynamics. The suggested methodology, making use of already computed parameters, is presented without derivation.

To compute the spacecraft position at time $t_{i}$, begin by computing the mean anomaly as

(143) $${\tilde{M}}_{i}=\sqrt{\frac{\mu }{a^{3}}}\left(t_{i}-t_{p_{0}}\right)-2\pi k_{i}$$

The tilde above $M_{i}$ is used to differentiate the mean anomaly found here from that found earlier (see Equation (141)). The value ${\tilde{M}}_{i}$ is computed by the best-fit orbit and elapsed time since the estimated $t_{p_{0}}$, while $M_{i}$ is computed from the best-fit orbit and the direction of the measured velocity vector at $t_{i}$. Given ${\tilde{M}}_{i}$, solve Kepler’s equation (see Equation (140)) to obtain ${\tilde{E}}_{i}$ (the solution to Kepler’s equation is necessarily iterative, with stable solutions found in most texts on this topic [187]), which may be used to find the true anomaly ${\tilde{\nu }}_{i}$,

(144) $$\sin\left({\tilde{\nu }}_{i}\right)=\frac{\sin\left({\tilde{E}}_{i}\right)\sqrt{1-e^{2}}}{1-e\cos({\tilde{E}}_{i})}$$

(145) $$\cos\left({\tilde{\nu }}_{i}\right)=\frac{\cos({\tilde{E}}_{i})-e}{1-e\cos({\tilde{E}}_{i})}$$

By construction (see Figure 14), observe that unit vector pointing from the central body to the spacecraft may be written directly in vector form as a function of true anomaly $\nu_{i}$

(146) $$\langle {\tilde{r}}_{sc_{i}}\rangle =\cos\left({\tilde{\nu }}_{i}\right)\;\langle e\rangle +\sin\left({\tilde{\nu }}_{i}\right)\left(k\times \langle e\rangle \right)$$

where, since e is computed from Equation (124), the term $\left(k\times \langle e\rangle \right)$ is guaranteed to also be a unit vector and need not be renormalized. The final missing piece may be found using the classical expression for the orbit radius

(147) $${\tilde{\rho }}_{sc_{i}}=∥r_{sc_{i}}∥=\frac{p}{1+e\cos({\tilde{\nu }}_{i})}$$

where *p* is the semilatus rectum, which may be written directly in terms of hodograph fit parameters by substituting from Equation (128),

(148) $$p=\frac{h^{2}}{\mu }=\frac{\mu }{R^{2}}$$

These results for $\langle {\tilde{r}}_{sc_{i}}\rangle$ and ${\tilde{\rho }}_{sc_{i}}$ allow for an improved solution for $r_{sc_{i}}$ by substitution into Equation (130).

### 6.3. Numerical Results

**Figure 15:**
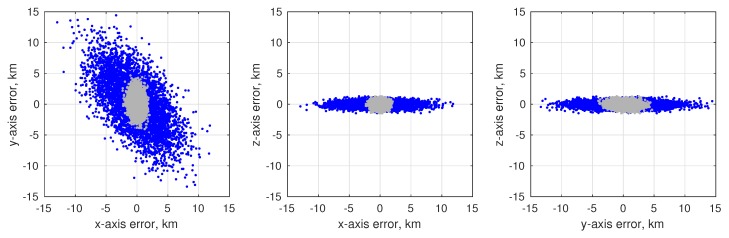
Example IOD residuals in position for a spacecraft in geostationary orbit. Blue dots show errors using method from [2] and gray dots show errors using improved method from this work.

**Figure 16:**
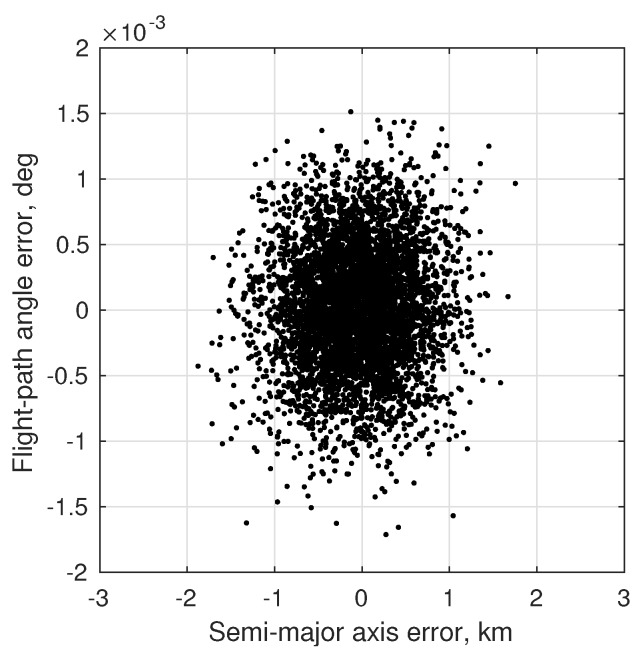
Example IOD residuals in semi-major axis and flight-path angle for a spacecraft in geostationary orbit.

The performance of velocity-only IOD using StarNAV measurements is demonstrated for an example spacecraft in GEO. Consider the situation where four stars are simultaneously viewed. Three of the stars are orthogonal to one another, and the fourth star is about 55 deg from each of the first three stars. Measurements to these stars are collected once every 10 min (600 s) with a 1σ error of 0.1 mas. Measurements are collected over one full orbit (24 h) resulting in a total of 144 StarNAV velocity estimates, which are computed using the algorithm from Section 5.3. The unknown position at the center time is computed using both the original method of [2] and the improved method from Section 6.2, with residuals for each shown in Figure 15. Additionally, for just the improved method, the residuals for the estimated semi-major axis and flight-path angle (FPA, $\gamma_{FPA}$) are shown in Figure 16. The semi-major axis may be computed directly from the hodograph fit parameters as (making use of Equations (124) and (148))

(149) $$a=\frac{p}{1-e^{2}}=\frac{\mu }{R^{2}-c^{T}c}$$

and the FPA may be computed from the obit fit and either ${\tilde{E}}_{i}$ or ${\tilde{\nu }}_{i}$.

**Figure 17:**
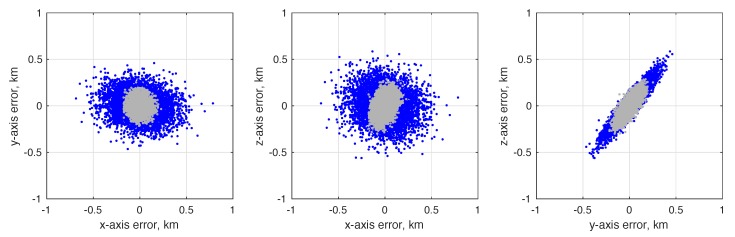
Example IOD residuals in position for a spacecraft in LEO. Blue dots show errors using method from [2] and gray dots show errors using improved method from this work.

**Figure 18:**
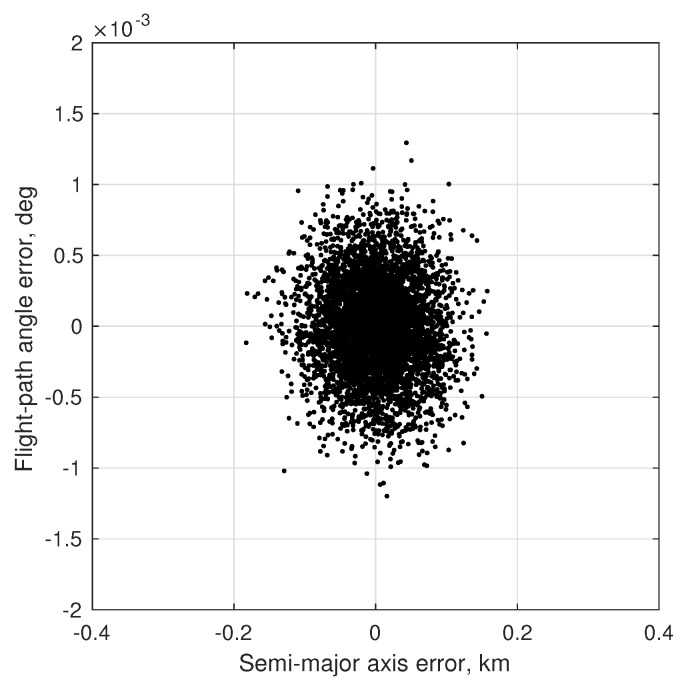
Example IOD residuals in semi-major axis and flight-path angle for a spacecraft in LEO.

Within the context of velocity-only IOD, the same StarNAV system generally performs better on faster orbits where the measurement noise represents a smaller percentage of the total velocity. For example, consider a spacecraft in a 410 km altitude LEO orbit at an inclination of 51.6 deg that collects measurements of the same quality as the preceding GEO example (four simultaneous stars with 1σ error of 0.1 mas). Assuming measurements are collected once every minute for an entire orbit (total of 93 measurements) the IOD position error at the middle time is as shown in Figure 17. The semi-major axis error and FPA error are shown in Figure 18. A comparison of LEO results (Figure 17 and Figure 18) with the GEO results (Figure 15 and Figure 16) highlights how the same system yields better IOD performance in a faster orbit.

## 7. Sequential Processing of StarNAV Observables with an Extended Kalman Filter

Once an initial orbit estimate is obtained—either via StarNAV IOD (see Section 6) or any other means of IOD (e.g., GPS, ground-based tracking, celestial OPNAV, XNAV)—it is more appropriate to process new StarNAV measurements as they become available within a sequential estimation framework. Such sequential estimation tasks are usually achieved through one of the many variants of the Kalman filter [196,197].

This work follows the classical extended Kalman filter (EKF) framework summarized in [198,199], while making use of modern navigation filter best practices [200]. The following discussions summarize the EKF framework, special considerations regarding the adaptation of the StarNAV measurement model, and example numerical results.

### 7.1. EKF Framework

This section presents a simple proof-of-concept filter for a spacecraft orbiting a large central body. Design of a filter to navigate an actual spacecraft is considerably more nuanced, and would likely require a more sophisticated dynamics model and inclusion of additional mission-specific states—either as solve-for parameters, consider parameters, or dynamic model compensation (DMC) process noise terms. Therefore, this work presents a simple (but architecturally representative) filter to avoid confounding performance of the StarNAV technique with mission-specific challenges. Detailed performance studies for particular design reference missions (DRMs) are left to future work.

The specific filter implementation used here is laid out in detail within the following subsections. The detailed presentation is provided not because it is especially novel, but because it is necessary to provide complete transparency on the framework that produced the numerical results in Section 7.2.

The state observability from any single inter-star angle measurement is principally driven by the bisector of the two contributing star directions. The component of the velocity perpendicular to the bisector direction is (for all practical purposes) unobservable for the corresponding measurement. Thus, at least three inter-star angles are needed (with bisectors that are not collinear) for full observability, which is only achievable with direction measurements to at least three stars. For these reasons, the StarNAV EKF approach presented here assumes a system designed to observe three guide stars that are used to compute three inter-star angle measurements (similar in concept to the notional diagram in Figure 5).

#### 7.1.1. State Vector Selection

The simplest realistic filter requires a nine-element state vector, consisting of the 3DOF translational dynamic state (3×1 position and 3×1 velocity) along with a unique scalar bias for each measured star pair (three star pairs in this case)

(150) $$x=\left[\begin{array}{c} r_{sc}\\ v_{sc}\\ b \end{array}\right]$$

where $r_{sc}$ is the Earth-Centered Inertial (ECI) position, $v_{sc}$ is the ECI velocity, and b is the bias vector. The ECI coordinate frame axes are assumed to be aligned with ICRF (and BCRF) with origin translated to the center of the Earth. Thus, following the conclusions from Section 5.1, the spacecraft’s ECI position and velocity are related to their instantaneous SSB counterparts through a simple translation,

(151) $$r=r_{sc}+r_{E}$$

(152) $$v=v_{sc}+v_{E}$$

where $r_{E}$ is the BCRF position of the Earth and $v_{E}$ is the BCRF velocity of the Earth. The bias terms are modeled as a first-order Gauss-Markov (FOGM) process.

#### 7.1.2. State and Covariance Propagation

Following standard practice, the state is assumed to evolve according to nonlinear dynamics

(153) $$\dot{x}=f\left(x\left(t\right),t\right)+w\left(t\right)$$

where f(x(t),t) is the nonlinear dynamics function and w(t) is assumed to be Gaussian zero-mean process noise, E[w(t)]=0, with power spectral density (PSD) Q(t),

(154) $$E\left[w\left(t_{i}\right)w^{T}\left(t_{j}\right)\right]=Q\left(t_{i}\right)\delta \left(t_{i}-t_{j}\right)$$

and where $\delta (t_{i}-t_{j})$ is the Dirac delta function. Under such a model, the *a posteriori* state estimate at time $t_{k-1}$, ${\hat{x}}_{k-1}^{+}$, is propagated forward in time to become the *a priori* state estimate at time $t_{k}$, $x_{k}^{-}$, by

(155) $${\hat{x}}_{k}^{-}={\hat{x}}_{k-1}^{+}+\int_{t_{k-1}}^{t_{k}}f\left(\hat{x}\left(\tau \right),\tau \right)\;d\tau$$

Likewise, the state covariance, P, is propagated according to

(156) $$P_{k}^{-}=\Phi \left(t_{k},t_{k-1}\right)P_{k-1}^{+}\Phi^{T}\left(t_{k},t_{k-1}\right)+S_{k}$$

where $\Phi (t_{k},t_{k-1})$ is the state transition matrix (STM) from time $t_{k-1}$ to time $t_{k}$ and the process noise covariance matrix $S_{k}$ is

(157) $$S_{k}=\int_{t_{k-1}}^{t_{k}}\Phi \left(t_{k},\tau \right)Q\left(\tau \right)\Phi^{T}\left(t_{k},\tau \right)\;d\tau$$

Practical implementation requires tractable expressions for both $\Phi (t_{k},t_{k-1})$ and $S_{k}$. These are now developed for the specific problem at hand using the usual approach. Fortunately, propagation of the spacecraft dynamics ($r_{sc}$ and $v_{sc}$) happens somewhat separately from propagation of the sensor biases (b), which simplifies the following discussion.

Specifically, partition the STM and process noise covariance by components belonging to the dynamical state (subscript “s”) and the bias (subscript “b”) according to

(158) $$\Phi =\left[\begin{array}{cc} \Phi_{ss} & \Phi_{sb}\\ \Phi_{bs} & \Phi_{bb} \end{array}\right]$$

(159) $$S=\left[\begin{array}{cc} S_{ss} & S_{sb}\\ S_{bs} & S_{bb} \end{array}\right]$$

where, by construction, one finds that

(160) $$\Phi_{sb}=\Phi_{bs}^{T}=0_{6\times 3}$$

(161) $$S_{sb}=S_{bs}^{T}=0_{6\times 3}$$

Proceed, therefore, by first considering the terms for the dynamical states ($\Phi_{ss}$ and $S_{ss}$) and then considering the terms for the bias states ($\Phi_{bb}$ and $S_{bb}$).

Begin with the dynamical states. Assuming Keplerian motion, the position and velocity of the spacecraft relative to the central body may be propagated forward in time by integrating of the equations of motion [187]

(162) $${\dot{\hat{r}}}_{sc}={\hat{v}}_{sc}$$

(163) $${\dot{\hat{v}}}_{sc}=-\frac{\mu }{∥{\hat{r}}_{sc}{∥}^{3}}{\hat{r}}_{sc}$$

where $\mu =GM_{B}$ is the central body’s gravitational parameter. As this is a proof-of-concept filter, the detailed consideration of atmospheric drag, solar radiation pressure, gravitational perturbations (from both non-spherical potential of central body and third bodies), so-called unfortunate lack of acceleration knowledge (FLAK) events (e.g., venting on a crewed vehicle), and other similar real-world complications are deferred to future work.

The STM for the dynamical states, $\Phi_{ss}$, may be computed using any one of a variety of reasonable approximations suitable for use in practical filters [200,201,202]. This work makes use of one of the second-order methods proposed by Lear [201] for the numerous reasons outlined by Carpenter and D’Souza in [200]. This approximation is given by

(164) $$\Phi_{ss}\left(t_{k},t_{k-1}\right)=I+\frac{\Delta t}{2}\left(F_{{ss}_{k}}+F_{{ss}_{k-1}}\right)+\frac{\Delta t^{2}}{2}F_{{ss}_{k}}F_{{ss}_{k-1}}$$

where $\Delta t=t_{k}-t_{k-1}$ and $F_{ss}$ is the Jacobian of the dynamical states (which, for Keplerian dynamics, is a classical result found in most textbooks addressing spacecraft navigation [187,203,204])

(165) $$F_{ss}={\left.\frac{\partial f(x_{s}\left(t\right),t)}{\partial x_{s}}\right|}_{x_{s}={\hat{x}}_{s}}=\left[\begin{array}{cc} 0_{3\times 3} & I_{3\times 3}\\ G & 0_{3\times 3} \end{array}\right]$$

with

(166) $$G=-\frac{\mu }{∥{\hat{r}}_{sc}{∥}^{3}}I_{3\times 3}+\frac{3\mu }{∥{\hat{r}}_{sc}{∥}^{5}}{\hat{r}}_{sc}{\hat{r}}_{sc}^{T}$$

The process noise for the dynamical states are computed using a simple state noise compensation (SNC) model [200,203] that assumes a random walk in velocity caused by a white noise acceleration, $w_{v}$, such that the equations of motion for the dynamical states in Equation (153) become

(167) $${\dot{r}}_{sc}=v_{sc}$$

(168) $${\dot{v}}_{sc}=-\frac{\mu }{∥r_{sc}{∥}^{3}}r_{sc}+w_{v}$$

and the process noise $w_{v}$ is zero mean with a 3×3 PSD of $Q_{vv}$. Under these conditions, one finds that the 6×6 SNC process noise covariance for the dynamical states is [203]

(169) $$S_{ss}=\left[\begin{array}{cc} \frac{\Delta t^{3}}{3}Q_{vv} & \frac{\Delta t^{2}}{2}Q_{vv}\\ \frac{\Delta t^{2}}{2}Q_{vv} & \Delta tQ_{vv} \end{array}\right]$$

Attention is now shifted to consideration of the bias terms. As FOGM processes that have identical models but are uncorrelated with each other, the behavior of the bias parameters are governed by

(170) $$\dot{b}\left(t\right)=-\frac{1}{\tau }b\left(t\right)+w_{b}$$

where τ is the correlation time and $w_{b}$ is zero mean white noise with statistics

(171) $$w_{b}∼N\left(0_{3\times 1},\sigma_{w_{b}}^{2}I_{3\times 3}\right)$$

The value of τ is chosen to control how quickly the time correlation of the FOGM will fade.

One of the many desirable properties of FOGM parameters is that both the state and covariance may be propagated analytically,

(172) $${\hat{b}}_{k}^{-}=\exp\left(-\Delta t/\tau \right){\hat{b}}_{k-1}^{+}$$

such that the STM becomes

(173) $$\Phi_{bb}\left(t_{k},t_{k-1}\right)=\exp\left(-\Delta t/\tau \right)I_{3\times 3}$$

The corresponding process noise is computed as

(174) $$S_{bb}=\frac{\tau \sigma_{w_{b}}^{2}}{2}\left[1-\exp\left(-2\Delta t/\tau \right)\right]I_{3\times 3}$$

In summary, therefore, the STM is found by substituting Equations (160), (164) and (173) into Equation (158). Likewise, the process noise covariance is found by substituting Equations (161), (169) and (174) into Equation (159). This allows analytic advancement of the state covariance according to Equation (156).

#### 7.1.3. Measurement Update

The measurement update follows the conventional EKF approach. Assuming a nonlinear measurement model with additive Gaussian noise,

(175) $$y_{k}=h\left(x_{k}\right)+z_{k}$$

construct the measurement sensitivity matrix as the Jacobian of $h(x_{k})$

(176) $$H_{k}={\left.\frac{\partial h(x)}{\partial x}\right|}_{x={\hat{x}}_{k}^{-}}$$

Assuming a state update of the form

(177) $${\hat{x}}_{k}^{+}={\hat{x}}_{k}^{-}+K_{k}\left(y_{k}-h\left({\hat{x}}_{k}^{-}\right)\right)$$

the optimal solution is found when $K_{k}$ is the Kalman gain,

(178) $$K_{k}=P_{k}^{-}H_{k}^{T}{\left(H_{k}P_{k}^{-}H_{k}^{T}+R_{k}\right)}^{-1}$$

Finally, as has become standard practice due to numerical stability [200,204], the covariance is updated using the Joseph form [205] to help ensure the covariance remains symmetric and positive definite

(179) $$P_{k}^{+}=\left(I-K_{k}H_{k}\right)P_{k}^{-}{\left(I-K_{k}H_{k}\right)}^{T}+K_{k}R_{k}K_{k}^{T}$$

When using a set of inter-star angles formed from concurrent star sightings, the measurement covariance $R_{k}$ is the same as for the instantaneous velocity fix with inter-star angles (see Section 5.3). The primary difficulty lies in the appropriate construction of $H_{k}$ for use in the usual EKF measurement update equations. This requires some care and is now discussed in more detail.

Let the measurement vector be comprised of the independent inner products of the observed stars along with a measurement bias. For example, when three stars are observed, define $h(x_{k})$ as

(180) $$h\left(x_{k}\right)=\left[\begin{array}{c} h_{ij}\\ h_{i\ell }\\ h_{j\ell } \end{array}\right]=\left[\begin{array}{c} {u_{i}^{\prime \prime }}^{T}u_{j}^{\prime \prime }\\ {u_{i}^{\prime \prime }}^{T}u_{\ell }^{\prime \prime }\\ {u_{j}^{\prime \prime }}^{T}u_{\ell }^{\prime \prime } \end{array}\right]+\left[\begin{array}{c} b_{ij}\\ b_{i\ell }\\ b_{j\ell } \end{array}\right]=\left[\begin{array}{c} \cos\theta_{ij}^{\prime \prime }\\ \cos\theta_{i\ell }^{\prime \prime }\\ \cos\theta_{j\ell }^{\prime \prime } \end{array}\right]+\left[\begin{array}{c} b_{ij}\\ b_{i\ell }\\ b_{j\ell } \end{array}\right]$$

and $\cos\theta_{ij}^{\prime \prime }$ is from Equation (45).

Proceed by partitioning $H_{k}$ into the components belonging to each part of the state vector

(181) $$H_{k}=\left[\begin{array}{ccc} H_{r_{k}} & H_{v_{k}} & H_{b_{k}} \end{array}\right]$$

where

(182) $$H_{r_{k}}={\left.\frac{\partial h(x)}{\partial r_{sc}}\right|}_{x={\hat{x}}_{k}^{-}}\;\;\;H_{v_{k}}={\left.\frac{\partial h(x)}{\partial v_{sc}}\right|}_{x={\hat{x}}_{k}^{-}}\;\;\;H_{b_{k}}={\left.\frac{\partial h(x)}{\partial b}\right|}_{x={\hat{x}}_{k}^{-}}\;$$

The partial for $H_{b_{k}}$ is readily found as

(183) $$H_{b_{k}}=\frac{\partial b}{\partial b}=I_{3\times 3}$$

Expressions for $H_{r_{k}}$ and $H_{v_{k}}$ are most easily found by considering a single row at a time. That is, one has the scalar equation

(184) $$h_{ij}\left(x_{k}\right)=\cos\theta_{ij}^{\prime \prime }+b_{ij}$$

from which the following may be computed

(185) $$\frac{\partial h_{ij}\left(x\right)}{\partial r_{sc}}=\frac{\partial \cos\theta_{ij}^{\prime \prime }}{\partial u_{i}^{\prime }}\frac{\partial u_{i}^{\prime }}{\partial r}\frac{\partial r}{\partial r_{sc}}+\frac{\partial \cos\theta_{ij}^{\prime \prime }}{\partial u_{j}^{\prime }}\frac{\partial u_{j}^{\prime }}{\partial r}\frac{\partial r}{\partial r_{sc}}$$

(186) $$\frac{\partial h_{ij}\left(x\right)}{\partial v_{sc}}=\frac{\partial \cos\theta_{ij}^{\prime \prime }}{\partial v}\frac{\partial v}{\partial v_{sc}}$$

The only task remaining is to compute the necessary partials. Beginning with the nonlinear expression for $\cos\theta_{ij}^{\prime \prime }$ from Equation (45), one finds

(187) $$\frac{\partial \cos\theta_{ij}^{\prime \prime }}{\partial u_{i}^{\prime }}=\left(1-\cos\theta_{ij}^{\prime \prime }\right)\left[{\left(1-{u_{i}^{\prime }}^{T}u_{j}^{\prime }\right)}^{-1}{u_{j}^{\prime }}^{T}+{\left(1+\beta^{T}u_{i}^{\prime }\right)}^{-1}\beta^{T}\right]$$

(188) $$\frac{\partial \cos\theta_{ij}^{\prime \prime }}{\partial v}=\frac{\left(1-\cos\theta_{ij}^{\prime \prime }\right)}{c}\left[{\left(1+\beta^{T}u_{i}^{\prime }\right)}^{-1}{u_{i}^{\prime }}^{T}+{\left(1+\beta^{T}u_{j}^{\prime }\right)}^{-1}{u_{j}^{\prime }}^{T}+2{\left(1-\beta^{T}\beta \right)}^{-1}\beta^{T}\right]$$

Likewise, from the nonlinear expression for $u_{i}^{\prime }$ from Equations (29) and (33), one may compute the partial

(189) $$\begin{array}{c} \frac{\partial u_{i}^{\prime }}{\partial r}=-\sum_{B}\frac{2GM_{B}}{c^{2}{∥d_{iB}∥}^{2}}\left\{\left(1+u_{i}^{T}u_{B}\right)\left[I_{3\times 3}-2\langle d_{iB}\rangle {\langle d_{iB}\rangle }^{T}\right]{\left[u_{i}\times \right]}^{2}-\frac{1}{\rho_{B}}\left(d_{iB}u_{i}^{T}\right)\left(I_{3\times 3}-u_{B}u_{B}^{T}\right)\right\} \end{array}$$

where general relativity has been assumed such that $(\gamma_{PPN}+1)=2$. Neglecting terms of $O(c^{-2})$ and higher leads to considerable simplification of these partials for practical computation

(190) $$\frac{\partial \cos\theta_{ij}^{\prime \prime }}{\partial u_{i}^{\prime }}=\left(1-u_{i}^{T}u_{j}\right)\left[{\left(1-u_{i}^{T}u_{j}\right)}^{-1}u_{j}^{T}+\beta^{T}\right]+O\left(c^{-2}\right)$$

(191) $$\frac{\partial \cos\theta_{ij}^{\prime \prime }}{\partial v}=\frac{\left(1-u_{i}^{T}u_{j}\right)}{c}\left(u_{i}^{T}+u_{j}^{T}\right)+O\left(c^{-2}\right)$$

(192) $$\frac{\partial u_{i}^{\prime }}{\partial r}=0_{3\times 3}+O\left(c^{-2}\right)$$

Furthermore, the partials for transitioning from BCRF to ECI may be computed from Equations (151) and (152),

(193) $$\frac{\partial r}{\partial r_{sc}}=I_{3\times 3}\;\;\;\frac{\partial v}{\partial v_{sc}}=I_{3\times 3}$$

Consequently, for the three-star example from Equation (180), one finds the partitioned elements of $H_{k}$ to be

(194) $$H_{r_{k}}=0_{3\times 3}$$

(195) $$H_{v_{k}}=\frac{1}{c}\left[\begin{array}{c} \left(1-u_{i}^{T}u_{j}\right)\left(u_{i}^{T}+u_{j}^{T}\right)\\ \left(1-u_{i}^{T}u_{\ell }\right)\left(u_{i}^{T}+u_{\ell }^{T}\right)\\ \left(1-u_{j}^{T}u_{\ell }\right)\left(u_{j}^{T}+u_{\ell }^{T}\right) \end{array}\right]$$

(196) $$H_{b_{k}}=I_{3\times 3}$$

### 7.2. Numerical Results

As a representative example, consider a spacecraft in a 410 km altitude circular Earth orbit with an inclination of 51.6 deg. Measurements are taken every 10 s to three stars separated by 100 deg from each other. Individual star bearing measurements are assumed to have an error $\sigma_{\phi_{i}}=0.1$ mas, with a fully correlated inter-star measurement covariance given by Equations (113) and (114). Furthermore, each star has an unknown (but fixed) bearing bias of about 1 arcsec. Since the Gaia Data Release 2 catalog has star directions with errors on the order of 0.1 mas, this is equivalent to a 1 arcsec misalignment between the sensor components measuring the direction to each star. Thus the star direction measurements are assumed to have a precision of about 0.1 mas and an accuracy of about 1 arcsec (four orders of magnitude difference between the two). The three corresponding inter-star angles are computed and used as the measurement within the EKF.

The truth model includes the gravitational deflection of starlight, considering the effects of the Sun, Earth, Moon, and Jupiter. The positions of the planets are assumed known from the ephemeris files maintained by NASA’s Navigation and Ancillary Information Facility (NAIF) using the SPICE Toolkit [182,206]. As a consequence, the errors from in the gravitational deflection of light come primarily from errors in the estimate of the spacecraft position. Steady state position errors are better than 50 m (1σ), which results in a bearing error well below the noise floor.

Process noise used in this analysis follows the simple model of $Q_{vv}=qI_{3\times 3}$, with *q* being constant throughout the simulation. Results shown here assume $q=10^{-6}$ m${}^{2}$/s${}^{3}$.

**Figure 19:**
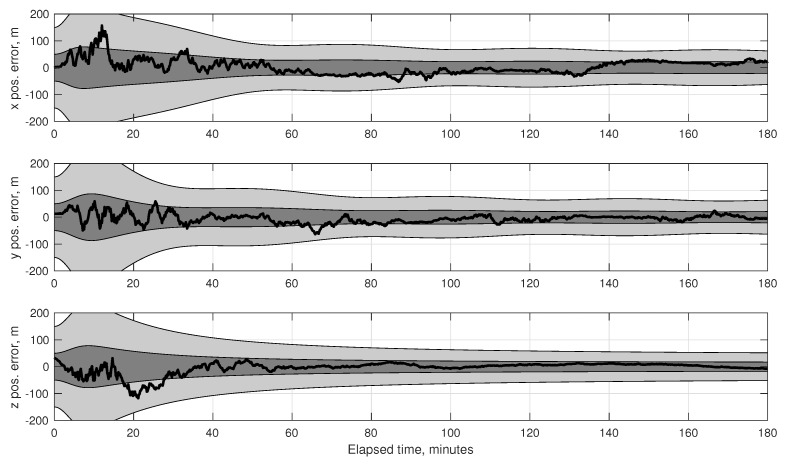
EKF position residuals for a spacecraft in a 410 km altitude circular Earth orbit with StarNAV measurements taken once every 10 s. Filter covariance is shown by shaded region (1σ in dark gray, 3σ in light gray).

**Figure 20:**
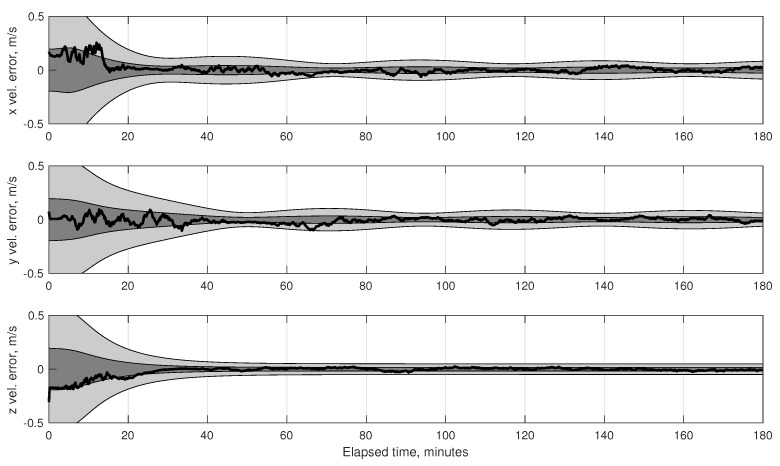
EKF velocity residuals for a spacecraft in a 410 km altitude circular Earth orbit with StarNAV measurements taken once every 10 s. Filter covariance is shown by shaded region (1σ in dark gray, 3σ in light gray).

Filter performance for such a situation is shown in Figure 19 and Figure 20. That the filter still works well with 1 arcsec of misalignment in each star direction provides empirical evidence that such biases are observable. Furthermore, the steady state velocity error is 4 cm/s, which is about 1/4 of the error associated with an instantaneous velocity fix with 0.1 mas star measurements. This highlights the performance improvement realized by filtering.

## 8. Conclusions

This work presents the StarNAV concept for using the relativistic perturbation of starlight to navigate a spacecraft anywhere in the Solar System (or, perhaps, beyond). The velocity of the spacecraft causes a change in the apparent wavelength (relativistic Doppler effect) and direction (stellar aberration) of a star as seen by sensor aboard the spacecraft. Thus, velocity may be estimated by measuring these changes—and these velocity estimates may be used for autonomous navigation.

This work shows that using the relativistic Doppler effect (the StarNAV-DE method) is likely impractical due to instability in stellar spectra and challenges with sensor technology. Furthermore, while the absolute change in star direction from stellar aberration is likely unobservable to the necessary precision in practice, measuring the change in the angle between two stars appears to be feasible. Thus, the StarNAV-SA method uses exclusively the perturbation in inter-star angle for navigation.

After development of measurement models and the mathematics necessary for an instantaneous velocity fix, the efficacy of StarNAV-SA is explored within the context of initial orbit determination (IOD) and an on-board sequential filter. Numerical results indicate that reasonable navigation performance may be achieved with existing technology. Substantial forward work remains, however, in the detailed engineering design of a StarNAV sensor system.

## Figures and Tables

**Table 1:** Photon flux as measured by a typical CCD detector for stars of type F and G.

Apparent Visual Magnitude, $m_{V}$	Number of Stars * Brighter than $m_{V}$	Photon Flux photons/m${}^{2}$/second
0	4	$2.741\times 10^{10}$
1	15	$1.091\times 10^{10}$
2	49	$4.344\times 10^{9}$
3	170	$1.729\times 10^{9}$
4	512	$6.885\times 10^{8}$
5	1601	$2.741\times 10^{8}$
6	5011	$1.091\times 10^{8}$

* Number of stars (of all types) from Version 2 of the Hipparcos catalog [97].

**Table 2:** Exclusion angles required to guarantee neglected star bearing perturbations remain below 0.01 mas for a spacecraft in GEO.

Celestial Body	$\theta_{\mathrm{exc}1}$	$\theta_{\mathrm{exc}2}$
Sun	179.7 deg	13.1 deg
Earth	154.0 deg	154.0 deg
Moon	0.75 deg	0.75 deg
Jupiter	11.2 deg	2.90 arcsec
Saturn	1.55 deg	0.18 arcsec

## Abbreviations

The following abbreviations are used in this manuscript:

BCRF	Barycentric Celestial Reference Frame
CCD	Charged Couple Device
DSAC	Deep Space Atomic Clock
DSN	Deep Space Network
EKF	Extended Kalman Filter
FOGM	First Order Gauss-Markov
FPA	Flight Path Angle
GEO	Geostationary Orbit
GNSS	Global Navigation Satellite Systems
IOD	Initial Orbit Determination
LEO	Low Earth Orbit
LOS	Line of Sight
OPD	Optical Path Delay
OPNAV	Optical Navigation
PPN	Parameterized Post-Newtonian
SNR	Signal-to-Noise Ratio
SSB	Solar System Barycenter
STM	State Transition Matrix
XNAV	X-ray Pulsar Navigation

## Appendix A. Historical Remarks on the Lorentz Transformation and Stellar Aberration

The consequences of the Lorentz transformation are numerous and its history is so tightly coupled with the problem of stellar aberration that a few historical notes are warranted. While central to the Special Theory of Relativity, the Lorentz transformation was already widely accepted by the time Einstein published [104] in 1905. Motivated by a number of puzzling problems—namely stellar aberration together with the results of the Michelson-Morley experiment and the Fizeau experiment—the 1890s witnessed a remarkable progression of thought that formed the backdrop for the development of special relativity. Of note is a series of developments by Lorentz during 1892–1895 [208,209,210] in which he suggested the concepts of length contraction (which was qualitatively described by FitzGerald a few years prior in 1889 [211]) and of local time.

These efforts paved the way for (nearly) concurrent development of the complete Lorentz transformation by both Lorentz (1899) [212] and Larmor (1900) [213], which were ultimately put into modern form and given the name *Lorentz Transformation* by Poincaré in 1905 [214]. At the time, however, Lorentz, Larmor, and Poincaré all maintained belief in the aether. It was Einstein who first derived this transformation from first principles—and, in doing so, removed reliance on the artificial construct of the aether and finally provided a satisfying answer to the problem of stellar aberration (and, notably, explanations of the results from the Michelson-Morley experiment and the Fizeau experiment).

The Lorentz transformation equations for describing an object’s coordinate {x,y,z,t} in two frames (*S* and $S^{\prime }$), where the frame $S^{\prime }$ is moving relative to frame *S* with a velocity *v* along the *x*-axis only, may be found in almost any introductory physics textbook (e.g., [215]),

(A1a) $$x^{\prime }=\gamma \left(x-vt\right)$$

(A1b) $$y^{\prime }=y$$

(A1c) $$z^{\prime }=z$$

(A1d) $$t^{\prime }=\gamma \left(t-vx/c^{2}\right)$$

where γ is the Lorentz factor,

(A2) $$\gamma =1/\sqrt{1-v^{2}/c^{2}}$$

Since the present application is for autonomous navigation, it is often more convenient to rewrite the standard textbook version in vector form. Thus, decomposing the position r into components parallel and perpendicular to the velocity vector, one has

(A3) $$r_{∥}=\left(\langle v\rangle {\langle v\rangle }^{T}\right)r\;\;\;r_{⊥}=\left(I_{3\times 3}-\langle v\rangle {\langle v\rangle }^{T}\right)r$$

where $\langle \cdot \rangle$ denotes vector length normalization. The Lorentz transformation is then

(A4a) $$r_{∥}^{\prime }=\gamma \left(r_{∥}-vt\right)$$

(A4b) $$r_{⊥}^{\prime }=r_{⊥}^{\prime }$$

(A4c) $$t^{\prime }=\gamma \left(t-\left(v^{T}r\right)/c^{2}\right)$$

with

(A5) $$\gamma =1/\sqrt{1-\left(v^{T}v\right)/c^{2}}$$

Stellar aberration follows directly from this transformation. Suppose there is a photon with velocity w=dr/dt as seen by an observer in frame *S*, and with a velocity $w^{\prime }=dr^{\prime }/dt^{\prime }$ as seen by an observer in frame $S^{\prime }$. These two may be related by first taking the differential of the the Lorentz transformation,

(A6a) $$dr_{∥}^{\prime }=\gamma \left(dr_{∥}-vdt\right)$$

(A6b) $$dr_{⊥}^{\prime }=dr_{⊥}$$

(A6c) $$dt^{\prime }=\gamma \left(dt-\left(v^{T}dr\right)/c^{2}\right)$$

such that

(A7) $$w^{\prime }=\frac{dr^{\prime }}{dt^{\prime }}=\frac{dr_{∥}^{\prime }+dr_{⊥}^{\prime }}{dt^{\prime }}=\frac{\gamma \left(dr_{∥}-vdt\right)+dr_{⊥}}{\gamma (dt-\left(v^{T}dr\right)/c^{2})}$$

Simple algebraic rearrangement quickly shows that

(A8) $$dt^{\prime }=\gamma dt\left(1-\left(v^{T}w\right)/c^{2}\right)$$

and, therefore,

(A9) $$w^{\prime }=\frac{\gamma \left(w_{∥}-v\right)+w_{⊥}}{\gamma (1-\left(v^{T}w\right)/c^{2})}$$

which is the same as

(A10) $$w^{\prime }=\frac{w+\left(\gamma -1\right)\langle v\rangle {\langle v\rangle }^{T}w-\gamma v}{\gamma (1-\left(v^{T}w\right)/c^{2})}$$

Now, since w and $w^{\prime }$ are the velocity of a photon seen by two different observers, they may be written in terms of the line-of-sight direction from the observer to the source: w=−cu and $w^{\prime }=-cu^{\prime }$. Substituting this result,

(A11) $$-cu^{\prime }=\frac{-cu+\left(\gamma -1\right)\langle v\rangle {\langle v\rangle }^{T}\left(-cu\right)-\gamma v}{\gamma (1+\left(v^{T}u\right)/c)}$$

which becomes

(A12) $$u^{\prime }=\frac{1}{\gamma (1+v^{T}u/c)}\left[u+\left(\gamma -1\right)\langle v\rangle {\langle v\rangle }^{T}u+\frac{\gamma }{c}v\right]$$

which is clearly the same as the expressions for stellar aberration presented in Equations (40)–(42).

Stellar aberration is often described in terms of the change in angle between the light ray’s tangent vector and the velocity vector. Therefore, define the angle ϕ as follows

(A13) $$\cos\left(\phi \right)={\langle v\rangle }^{T}\langle w\rangle =-{\langle v\rangle }^{T}u$$

(A14) $$\cos\left(\phi^{\prime }\right)={\langle v\rangle }^{T}\langle w^{\prime }\rangle =-{\langle v\rangle }^{T}u^{\prime }$$

Proceed by left multiplying Equation (A12) by $-{\langle v\rangle }^{T}$

(A15) $$-{\langle v\rangle }^{T}u^{\prime }=\frac{1}{\gamma (1+v^{T}u/c)}\left[-{\langle v\rangle }^{T}u-\left(\gamma -1\right){\langle v\rangle }^{T}u-\frac{\gamma }{c}{\langle v\rangle }^{T}v\right]$$

such that the expression my be rewritten in terms of cos(ϕ) by substitution of Equations (A13) and (A14),

(A16) $$\cos\left(\phi^{\prime }\right)=\frac{1}{\gamma (1-v\cos(\phi )/c)}\left[\cos\left(\phi \right)+\left(\gamma -1\right)\cos\left(\phi \right)-\frac{\gamma }{c}v\right]$$

which further simplifies to

(A17) $$\cos\left(\phi^{\prime }\right)=\frac{\cos(\phi )-v/c}{1-\cos(\phi )v/c}$$

This expression for stellar aberration is identical to the result presented by Einstein on p. 912 of his original 1905 paper on special relativity [104].

Finally, noticing that ∥β∥=v/c

(A18) $$\cos\left(\phi^{\prime }\right)=\frac{\cos(\phi )-∥\beta ∥}{1-∥\beta ∥\cos(\phi )}$$

which is equivalent to that of Equation (57).
